# Polyethylenimine, an Autophagy-Inducing Platinum-Carbene-Based Drug Carrier with Potent Toxicity towards Glioblastoma Cancer Stem Cells

**DOI:** 10.3390/cancers14205057

**Published:** 2022-10-15

**Authors:** Conor McCartin, Candice Dussouillez, Chloé Bernhard, Eric Mathieu, Juliette Blumberger, Monique Dontenwill, Christel Herold-Mende, Ahmed Idbaih, Philippe Lavalle, Stéphane Bellemin-Laponnaz, Antoine Kichler, Sylvie Fournel

**Affiliations:** 1 3Bio Team, CAMB UMR7199 CNRS, Faculté de Pharmacie, University of Strasbourg, 67401 Ilkirch, France; 2 Laboratoire de Bioimagerie et Pathologies UMR CNRS 7021 (LBP), Faculté de Pharmacie, University of Strasbourg, 67401 Illkirch, France; 3 Institut National de la Santé et de la Recherche Médicale, Inserm UMR_S 1121 Biomaterials and Bioengineering, 67085 Strasbourg, France; 4 Faculté de Chirurgie Dentaire de Strasbourg, University of Strasbourg, 67000 Strasbourg, France; 5 Division of Neurosurgical Research, Department of Neurosurgery, University of Heidelberg, 69117 Heidelberg, Germany; 6 AP-HP, Institut du Cerveau—Paris Brain Institute—ICM, Inserm, CNRS, Hôpitaux Universitaires La Pitié Salpêtrière—Charles Foix, DMU Neurosciences, Service de Neurologie 2-Mazarin, Sorbonne University, 75013 Paris, France; 7 Institut de Physique et Chimie des Matériaux de Strasbourg (IPCMS) UMR7504 CNRS, University of Strasbourg, 67034 Strasbourg, France

**Keywords:** polymer–drug conjugate, polyethylenimine, N-heterocyclic carbene, platinum, cancer stem cells, autophagy, glioblastoma

## Abstract

**Simple Summary:**

The resistance of tumours to treatment and their tendency to reoccur following successful treatment are major problems for long-term cancer patient survival. Research has identified a special subset of cells in tumours with stem-cell-like properties to be at the heart of this problem. Thus, the development of new chemotherapeutic compounds with unique characteristics that may target this subset of cancer cells is of great interest in the pursuit of more effective cancer treatments. This work describes one such compound, being a new platinum-based compound attached to a long polymer molecule in order to enhance its anti-cancer properties. Importantly, the stem-cell-like subset of cells seems to be considerably more sensitive to the toxicity of the polymer itself than the bulk of tumour cells, with an interesting mode of cell death which has characteristics different to that of more “classical” anti-cancer therapeutics.

**Abstract:**

The difficulty involved in the treatment of many tumours due to their recurrence and resistance to chemotherapy is tightly linked to the presence of cancer stem cells (CSCs). This CSC sub-population is distinct from the majority of cancer cells of the tumour bulk. Indeed, CSCs have increased mitochondrial mass that has been linked to increased sensitivity to mitochondrial targeting compounds. Thus, a platinum-based polyethylenimine (PEI) polymer–drug conjugate (PDC) was assessed as a potential anti-CSC therapeutic since it has previously displayed mitochondrial accumulation. Our results show that CSCs have increased specific sensitivity to the PEI carrier and to the PDC. The mechanism of cell death seems to be necrotic in nature, with an absence of apoptotic markers. Cell death is accompanied by the induction of a protective autophagy. The interference in the balance of this pathway, which is highly important for CSCs, may be responsible for a partial reversion of the stem-like phenotype observed with prolonged PEI and PDC treatment. Several markers also indicate the cell death mode to be capable of inducing an anti-cancer immune response. This study thus indicates the potential therapeutic perspectives of polycations against CSCs.

## 1. Introduction

Platinum compounds have been an integral part of the chemotherapeutic arsenal against cancer since the approval of cisplatin in the 1970s [1], with an estimated 28% of cancers being treated with a platinum-based therapeutic [2]. The current understanding of their known mechanisms indicates a general activation of apoptosis through the accumulation of DNA damage, which, along with the development of these compounds, has been extensively reviewed [3,4,5]. The clinical success of cisplatin has led chemists to diversify the ligands around the platinum to improve the efficacy of cisplatin while reducing its side effects. In the last decade, N-heterocyclic carbene (NHC) as a platinum stabilising ligand has demonstrated new possibilities in medicinal chemistry [6,7]. Indeed, NHC-Pt complexes exhibit superior cytotoxic activities to cisplatin or related compounds, along with high stability.

However, they still present some drawbacks such as their biocompatibility. Among the challenges in the further development and improvement of small molecule anti-cancer treatments, such as NHC-Pt molecules, are their aqueous solubility and targeted delivery/release to tumour sites [8]. This can be addressed through the development of polymer–drug conjugates (PDCs), whereby drugs with otherwise poor biological solubility may be improved by their chemical conjugation to a large hydrophilic polymer [8]. This may also have the added benefit of the potential physicochemical reformation of the disordered polymer into a nanoparticle through the organisation of the conjugated hydrophobic small molecules into a water-excluded core [8,9,10,11]. Such a trait is highly desirable due to the supposed specific accumulation of nanoparticles within solid tumours due to the enhanced permeability and retention (EPR) effect [12,13].

Polymers which have been described for the synthesis of PDCs include polyethylene glycol (PEG), dextran, N-(2-hydroxypropyl)methacrylamide (HPMA), and a variety of dendrimers [8]. With over a dozen (almost all of which are PEG-based) PDCs having market approval at the time of publishing for a variety of uses [8]. Polyethylenimine (PEI), a commonly used transfection reagent for the introduction of genetic material into mammalian cells, also has the potential as a delivery agent for drugs [14]. So far, it has mainly been described for the delivery of nucleic acids such as plasmid DNA [15] with several linear PEI-based formulations being evaluated in clinical trials [15]. However, PEI has also been used as a carrier for antitumoral drugs such as doxorubicin [16], camptothecin [17] and an NHC-platinum compound (which is elaborated in this work; [11,18]).

Of particular interest in the latest developments of the anti-cancer chemotherapeutic domain is the targeting of the so-called cancer stem cell (CSC) subpopulation (also referred to as tumour-initiating cells) [19]. These cells are characterised by their stem-like characteristics such as asymmetric division, allowing their self-renewal and differentiation [20,21,22]. This has led to their implication in the driving of tumour recurrence due to their known propensity to resist current chemotherapeutic treatments [23,24]. The drivers of this resistance are metabolic reprogramming relative to more differentiated tumour cells making up the bulk of the tumour mass, upregulated DNA repair, upregulated anti-apoptotic proteins and drug efflux proteins, as well as protective autophagy [25]. Autophagy is an intricate cellular mechanism used for the recycling or removal of unneeded or damaged intracellular components [26] through their sequestration (in autophagosomes) and subsequent hydrolysis via acidification of the compartment through lysosomal fusion [26], thus providing a source of nutrients such as in times of starvation or stress [27].

Among the cancers where a significant CSC population has been identified is glioblastoma [28,29]. It is a highly aggressive brain cancer, which despite an established treatment consisting of surgical resection, radiotherapy, and chemotherapy with the alkylating agent temozolomide [28], has a high rate of recurrence linked to CSCs [28], and a median survival of only 12–15 months following diagnosis [29]. Targetable characteristics of CSCs have thus come to the forefront in chemotherapeutic development, such as their observed sensitivity to mitochondrial targeting compounds linked to an upregulated mitochondrial mass [30,31,32,33] and their upregulation of markers such as CD133 and CD44 (hyaluronic acid receptor) which may serve as cell surface targets [34,35].

Additionally, the role of the adaptive immune response against cancer has become an important aspect when considering the action and efficacy of anti-cancer chemotherapeutics [36]. The ability of a molecule to effectively and directly eliminate cancer cells is made considerably more interesting if the mode of cell death may stimulate the adaptive immune response against the tumour in question, contributing to its elimination, and potentially safeguarding against its recurrence [36]. This so-called “immunogenic cell death” (ICD), implies the release of damage-associated molecular patterns (DAMPs) from dying cells [37]. DAMPs are normally intracellular factors which are capable of stimulating the innate immune response via interaction with pattern recognition receptors (PRRs). This leads to the recruitment and maturation of professional antigen-presenting cells (APCs) such as dendritic cells, which may also phagocytose the cells [36,38], present tumour-specific or associated antigens, and thus initiate an anti-tumour T-cell response. DAMPs, of which there are several types with varying effects, include outer-membrane exposure of the normally endoplasmic reticulum localised protein calreticulin, and ATP, which serves as an immunostimulatory compound via binding to purinergic receptors which stimulate the production of pro-inflammatory cytokines [36,39].

The aforementioned NHC-platinum(II) PDC, a complex shown to form nanoparticles in aqueous solution, named NHC-Pt(II)-PEI, has been developed by our team and has already proven to be effective against several human and murine cancer cell lines in vitro and in vivo using a nude (immunodeficient) mouse tumour model [11,18]. The anti-tumour efficacy of the PDC and its accumulation within the mitochondria, associated with mitochondrial dysfunction of HCT116 cells shown in the same study led us to the hypothesis that the compound may present an even higher level of toxicity against CSCs. Thus, in this study, we wished to evaluate whether the PDC or indeed the polymer carrier itself, PEI, presented a specifically high level of toxicity towards CSCs. We also wished to evaluate the characteristics of the cell death induced, and the potential for induction of ICD.

## 2. Materials and Methods

### 2.1. Storage of Compounds

NHC-Pt(II)-PEI (22 kDa PEI with one NHC-Pt(II) coordinated every 30 repeating units; thus, 17 NHC-Pt(II), for a total molecular mass of ~30.2 kDa) and 22 kDa PEI were stored as stock solutions in absolute ethanol. Oxaliplatin (#O9512, Sigma-Aldrich, Saint-Quentin-Fallavier, France), chloroquine (#PHR1258, Sigma-Aldrich, Saint-Quentin-Fallavier, France) and 30 kDa poly-L-Lysine (#P9404, Sigma-Aldrich, Saint-Quentin-Fallavier, France) were stored as solutions in MilliQ H_2_O, while all other compounds used were stored as solutions in DMSO. Stocks were stored at −20 °C or −80 °C. Prior to use, stocks were heated and sonicated briefly at 37 °C in a bath sonicator. Prior to the treatment of cells, stock compounds were diluted to desired concentrations in the culture medium of the cells being treated. The NHC-Pt(II)-PEI PDC was synthesised as described by Chekkat et al. (2016) [11]. The linear 22 kDa PEI was synthesised according to Brissault et al., 2003 [40].

### 2.2. Cell Culture and Treatment Conditions

#### 2.2.1. U87-MG Cells

U87-MG (HTB-14, ATCC, Manassas, VA, USA) cells were obtained from the M. Dontenwill lab (UMR 7021 CNRS, University of Strasbourg, Faculty of Pharmacy, Illkirch, France). The cells were maintained in regular tissue culture treated T75 flasks (#658170, Greiner Bio-One, Les Ulis, France) containing Roswell Park Memorial Institute (RPMI) 1640 medium (#R2405-500 ML, Sigma-Aldrich, Saint-Quentin-Fallavier, France) medium containing 10% (*v/v*) fetal bovine serum (FBS) (#10270106, Gibco, Waltham, MA, USA), Penicillin-Streptomycin (P/S: 10 U/0.1 mg) (#P0781-100ML, Sigma-Aldrich, Saint-Quentin-Fallavier, France) in a 37 °C, 80% humidity and 5% CO_2_ incubator. Once confluence reached ~80%, cultures were maintained by removal of spent culture medium, washing twice with Dulbecco’s Phosphate Buffered Saline (PBS) (#D8537-500ML, Sigma-Aldrich, Saint-Quentin-Fallavier, France), followed by treatment with trypsin-EDTA (at 0.5 mg/mL and 0.2 mg/mL, respectively) (#T3924-100ML, Sigma-Aldrich, Saint-Quentin-Fallavier, France) for 5 min at 37 °C, collection of cells, washing, resuspension in culture medium and re-seeding in a new T75 flask. Cells were counted via trypan blue (#T8154-100ML, Sigma-Aldrich, Saint-Quentin-Fallavier, France) exclusion using the Countess II FL automated cell counter (Thermo Fisher Scientific, Illkirch-Graffenstaden, France). For treatment, unless otherwise stated, cells were seeded at 18,000 cells per well in 100 µL medium in a flat-bottomed tissue culture treated 96-well culture plate (#655180, Greiner Bio-One, Les Ulis, France). Treatment was applied by aspiration of culture medium from wells and replacement with the desired dilutions of the compounds.

#### 2.2.2. Dental Pulp Stem Cells

Dental pulp stem cells (DPSCs) were obtained from the Pr. Florent Meyer lab (INSERM 1121, Faculté de Chirurgie Dentaire, Université de Strasbourg, Strasbourg, France). The cells were maintained in regular tissue culture treated T75 flasks using Minimum Essential Medium (MEM) Alpha Medium (1×) + Glutamax (#32561-029, Gibco, Illkirch-Graffenstaden, France) medium containing 10% (*v/v*) FBS, Penicillin-Streptomycin (P/S; 10 U/0.1 mg) in a 37 °C, 80% humidity and 5% CO_2_ incubator. Cells were passaged and counted in the same manner as the U87-MG cells. For treatment, unless otherwise stated, cells were seeded at 25,000 cells per well in 100µL medium in a U-bottomed 96-well suspension culture plate (#650185, Greiner Bio-One, Les Ulis, France) in order to encourage spheroid formation. Treatment was applied by the addition of 10µL medium containing the desired compound at 11× the desired final concentration.

#### 2.2.3. Glioblastoma Stem Cells

NCH421K and NCH644 glioblastoma CSCs (GSCs) were obtained from Pr. Christel Herold-Mende (Division of Neurosurgical Research, Department of Neurosurgery, University of Heidelberg, Germany) [41]. The 3731 GSCs were obtained from Dr. A. Idbaih's group (Sorbonne Université/AP-HP/ICM, Paris, France) [42]. GSCs growing as spheroids were maintained in regular tissue culture treated T25 (#690175, Greiner Bio-One, Les Ulis, France) or T75 cell culture flasks in CSC culture medium ((Dulbecco’s modified eagle medium (DMEM)/Ham’s F12 (1:1) (#D6421, Sigma-Aldrich, Saint-Quentin-Fallavier, France) containing 20% (*v*/*v*) BIT 100 supplement (#2043100, Provitro, Berlin, Germany), 20 ng/mL basic fibroblast growth factor (FGF-2) (#130-093-842, Miltenyi Biotec, Paris, France), 20 ng/mL epidermal growth factor (EGF) (#130-097-751, Miltenyi Biotec, Paris, France), P/S (10 U–0.1 mg) and GlutaMAX supplement (#35050061, Gibco, Illkirch-Graffenstaden, France) in a 37 °C, 80% humidity and 5% CO_2_ incubator. Spheroid cultures were maintained by passaging once a week via recuperation of spheroids, washing with PBS, followed by treatment with Accutase (#A6964-100ML, Sigma-Aldrich, Saint-Quentin-Fallavier, France) for 5 min at room temperature. Dissociated cells were then washed once, resuspended in culture medium, and counted in the same manner as the U87-MG cells. Cells were re-seeded at 35,000 viable cells per mL. For treatment, unless otherwise stated, GSCs were seeded at 75,000 viable cells per mL (100 µL per well) in a regular flat-bottom tissue culture treated 96-well plate and were then left to form spheres for four days prior to treatment with the tested compounds the following day. Treatment was applied in the same manner as for the DPSCs.

#### 2.2.4. RAW Macrophages

The murine macrophage cell line RAW 264.7 (TIB-71, ATCC) (originating from BALB/c mice cells transformed with Abelson leukaemia virus [43]) was used as a model for the induction of a pro-immune or anti-inflammatory response. For facility of passaging, cells were cultured normally as a semi-adherent culture in 10 cm^2^ suspension culture dishes at 37 °C, 5% CO_2_ and 80% humidity in 12 mL DMEM (#D0819-500ML, Sigma-Aldrich, Saint-Quentin-Fallavier, France) medium supplemented with 5% FBS 100 U/mL penicillin and 0.1 mg/mL streptomycin (#P0781-100ML, Sigma). Cells were passaged by gentle flushing of the culture medium to remove the cells, with cells then pelleted (200 g, 3 min, RT), resuspended in medium and counted (Section 2.2.1). Cells were re-seeded at 5 × 10^6^ cells/mL or 10 × 10^6^ cells/mL for 2 or 3 days of growth, respectively.

For experiments, RAW 264.7 cells were seeded the day before treatment at a density of 3 × 10^4^ cells per well (100µL medium volume) in regular adherent 96-well culture plates (#655180, Greiner Bio-One, Les Ulis, France) or 3 × 10^5^ cells per well (300µL medium volume) in 12-well plates (#665180, Greiner Bio-One, Les Ulis, France).

### 2.3. Cell Viability Assay

Cell culture viability was measured by using the CelltiterGlo 3D Cell Viability Assay (#G9681, Promega, Charbonnières-les-Bains, France), which measures the quantity of ATP via luciferase activity. This decrease could be correlated with a decrease in cell viability, cell metabolism, or cell number. Treated cells were left for the desired time point prior to the addition of 110 µL CelltiterGlo 3D reagent. Wells were then periodically flushed vigorously via pipetting in order to disaggregate spheres and encourage lysis prior to transfer to a white opaque 96-well culture plate (#236105, ThermoFisher, Illkirch-Graffenstaden, France) to prevent luminescence leakage between wells during relative luminescence units (RLU) counting using a plate reader (SP2000 Safas, Monaco). Results were expressed by the subtraction of background RLU (culture medium and assay reagent) and expression of viability relative to non-treated controls (considered as 100% viability). The percentage of viability was calculated using the following equation (Equation (1)):

Viability = [#RLU(Treatment)/#RLU(Non-treated)] × 100(1)

IC50 values were calculated using GraphPad Prism software from dose–response curves using non-linear regression (log(concentration) vs. %viability—variable slope (four parameters)).

### 2.4. Spheroid Formation Assay

In order to follow the effect of treatment on the spheroid formation capacity of the NCH421K cells, spheroid formation from individual cells was followed by the real-time microscopic assessment using Incucyte^®^ technology (Essen BioScience, Royston Hertfordshire, UK). In total, 3000 NCH421K cells/well were seeded in a 96-well plate in medium containing the desired concentration of treatment and cultured for 6 days in an incubator at 37 °C, 5% CO_2_. Cell surface area (mm^2^) of the spheroids was followed with an IncuCyte Zoom Live Cell Analysis system. Images were taken every 4 h, with the size of the spheres normalised to time 0 (thus single dissociated cells) and calculated using IncuCyte Zoom 2018A software.

### 2.5. Annexin V/Propidium Iodide Assay

Cell death and apoptosis were investigated by double staining with APC conjugated Annexin V (AnV), a protein that binds to phosphatidylserine, an inner leaflet membrane phospholipid which is externalised during apoptosis, and propidium iodide (PI), a membrane-impermeable DNA intercalating agent that enters cells which have lost membrane integrity and fluoresces only when bound to nucleic acids. Following treatment, the cells were recovered, and the spheroids dissociated via Accutase treatment, then washed in AnV binding buffer (10 mM HEPES, 140 mM NaCl, 2.5 mM CaCl_2_, pH 7.3–7.4). Triplicates were pooled and stained with APC-conjugated AnV (#640920, BioLegend, Amsterdam, Netherlands) diluted 1/100 with prepared AnV binding buffer which was incubated for 15 min at room temperature and sheltered from light. Cells were washed with AnV binding buffer and transferred to tubes containing 15 µM PI (#P4170-10MG, Sigma-Aldrich, Saint-Quentin-Fallavier, France), which were analysed immediately via flow cytometry (FACSCalibur, Becton Dickinson, Franklin Lakes, New Jersey, United States). APC-AnV fluorescence was detected in the FL4 channel while PI fluorescence was detected in the FL3 channel. Data were analysed using FlowJo v10 software.

### 2.6. Caspase 3/7 Activation Assay

Caspase 3/7 activation was investigated using the Caspase-Glo^®^ 3/7 Assay System (#G8210 & #G8091, Promega, Charbonnières-les-Bains, France), which functions via a pro-luminescent caspase 3/7 DEVD-aminoluciferin substrate, thus generating processible luciferase substrate, and luminescence, proportional to caspase activity. Following treatment, 110 µL reagent was added to each well, which was then mixed by vigorous pipetting. After 30 min incubation at room temperature, cell-lysate was transferred to an opaque 96-well plate, with luminescence then measured using a plate reader (SP2000 Safas, Monaco).

### 2.7. Transmission Electron Microscopy

In order to assess in detail, the morphological effects that treatment may have on the cells, transmission electron microscopy (TEM) was carried out on treated cells. Cells were treated at the desired time point and concentration in T175 flasks (#661175, Greiner Bio-One, Les Ulis, France) in 30 mL culture volume. The spheres/cells were recovered and washed once in PBS before dissociation via Accutase treatment. Cells were then washed twice in PBS before fixation in cold 2.5% glutaraldehyde (Euromedex, Souffelweyersheim, France) in 0.05 M sodium cacodylate (Euromedex, Souffelweyersheim, France) buffer at pH 7.4. for 2.5 h. The cells were then washed three times in cold 0.175 M sodium cacodylate buffer for 10 min. The samples were then post-fixed for 1.5 h in 1% osmium tetroxide in 0.15 M sodium cacodylate buffer. The samples were washed 3 times again in the same washing buffer and dehydrated in cold graded series of ethanol (30%, 50%, 70%, 95%) for 7 min each, then twice in cold absolute ethanol and once in RT absolute ethanol for 5 min each. The composition of Spurr resin used (Sigma-Aldrich) was the following: 5.90 g of NSA (Nonenyl succinic anhydride), 4.10 g of ERL 4221 (cycloaliphatic epoxide resin), 1.59 g of DER 736 (Poly(propylene glycol) diglycidyl ether) and 0.1 g of DMAE (Dimethylethanolamine) as an accelerator. The samples were transferred successively for 30 min in 1 vol Spurr resin/1 vol absolute ethanol, 30 min in 100% Spurr resin, and twice for 1 h in the same resin. Finally, the cells were included in 250 µL polypropylene tubes and left at RT for 24 h and put in a 60 °C oven for polymerisation for 48 h. Ultra-thin sections were performed using an automatic ultra-microtome Reichert Jung Ultracut E (Leica Microsystems, Wetzlar, Germany) equipped with a diamond knife and collected on 100 mesh formvar carbon-coated grids. They were stained with 5% uranyl acetate solution for 20 min. After rinsing, the grids were stained with 4% lead citrate solution for 10 min (All of these products were purchased from Euromedex, Souffelweyersheim, France). Finally, the sections were observed using a Hitachi H-7500 instrument (Hitachi High Technologies Corporation, Tokyo, Japan) operating with an accelerating voltage of 80 kV. The images were digitally recorded with an AMT Hamamatsu digital camera (Hamamatsu Photonics, Shizuoka, Japan).

### 2.8. LDH Release Assay

In order to measure cell membrane permeability via the release of the metabolic enzyme lactate dehydrogenase (LDH) into the culture medium, the Pierce LDH Cytotoxicity Assay Kit (#88953, Thermo Scientific, Illkirch-Graffenstaden, France) was used. Forty-five minutes before the desired time point, 11 µL of 10× Lysis Buffer was added to a non-treated well and mixed by vigorous pipetting to serve as a maximum LDH activity control. For adherent cells, 50 µL was then removed from each well and transferred to a flat bottomed 96-well plate, with 50 µL Reaction Mixture (prepared as per the kit’s instructions) added to each well and mixed. For non-adherent cells, the culture plate was centrifuged before careful removal of 50 µL medium in order to ensure that the cells were at the bottom of the plate. The mixture was then incubated for 30 min at room temperature protected from light before the addition of 50 µL Stop Solution. Absorbance was then measured at 490 nm and 680 nm using a plate reader (SP2000 Safas, Monaco). Background (680 nm) from the instrument was then subtracted from the values (490 nm), which were then expressed as a percentage of the maximum LDH activity following subtraction of the medium-only control.

### 2.9. Western Blot

#### 2.9.1. Cell Treatment, Lysis, and Protein Quantification

In order to assess the levels of the lipidated form of the protein microtubule-associated proteins 1A/1B light chain 3 (LC3), LC3-II (correlating with an accumulation of autophagosomes in the cells) and p62 (whose levels correlate inversely with autophagic flux/activation), Western blotting was carried out. Cells were treated at the desired time point and concentration in 6-well plates in a total volume of 3 mL. For LC3, all treatments were applied for 6 h except chloroquine which was treated for 3 h. For p62, all treatments were applied for 6 h. Cells were collected, washed once in PBS and either stored as pellets at −80 °C or lysed straight away. Cell lysis was carried out by resuspension of pellets in 100 µL Lysis Buffer (PBS/sodium tetrasodium pyrophosphate (Na_4_O_7_P_2_) 100 mM/sodium orthovanadate (Na_3_VO_4_) 1 mM/sodium fluoride (NaF) 100 mM/Triton 1%), the cells were then vortexed for 10 s and left to rest on ice for 10 min. This was repeated twice more before sonication of the cells in a water bath for 10 s. Three more rounds of vortexing and resting on ice were repeated as previously, with the lysate then centrifuged for 10 min at 13,000 g (4 °C). The supernatant was removed and retained with the pellet debris discarded. Protein content was determined using the Bicinchoninic Acid (BCA) assay (#UP95425, Interchim, Montluçon, France). A standard protein curve was made using Bovine Serum Albumin (BSA), with 25 µL protein sample added to each well of a 96-well plate, to which 200 µL BCA working solution (comprised of 50 volumes Solution A (BCA solution) for 1 volume of Solution B (4% Copper (II) Sulphate Pentahydrate)) was added and incubated at 37 °C for 30 min before absorbance measurement at 560 nm using a plate reader (SP200 Safas, Monaco).

#### 2.9.2. SDS-PAGE

Protein samples were prepared by diluting a quantity of lysate containing 20 µg of protein to a final volume of 30µL in MilliQ H_2_O, to which 10 µL of 4× NuPAGE™ LDS Sample Buffer (#1610747, Bio-Rad, Marnes-la-Coquette, France) was added, with the addition of 2 µL 1 M Dithiothreitol (DTT) (#EU0006-C, Euromedex, Souffelweyersheim, France). The tubes were sealed using clips and boiled for 10 min at 95 °C in a heating block in order to denature the protein. Tubes were then briefly spun to recover the full liquid volume, with the entirety of the sample then loaded onto a pre-cast 4–20% gradient SDS-PAGE gel (Mini-PROTEAN^®^ TGX™ Precast Protein Gels, 10-well, 50 µL, #4561094, Bio-Rad, Marnes-la-Coquette, France). A 5 µL protein ladder (PageRuler Prestained 10–180 kDa Protein Ladder, #26616, ThermoSFisher, Illkirch-Graffenstaden, France) was also loaded. The gel was run in 1× Tris/Glycine buffer (#1610734, Bio-Rad) with 0.1% (*v/v*) SDS (#1610416, Bio-Rad, Marnes-la-Coquette, France) at 100 V until fully resolved.

#### 2.9.3. Western Blot

Resolved gels were immediately transferred to 0.2 µm PVDF membrane using Trans-Blot Turbo Mini 0.2 µm PVDF Transfer Packs (#1704156, Bio-Rad, Marnes-la-Coquette, France) with the semi-dry Trans-Blot Turbo Transfer System semi-dry (#1704150EDU, Bio-Rad, Marnes-la-Coquette, France), which was wet using 20% EtOH 1× Tris-Gly transfer buffer and transferred for 10 min (1.3 A up to 24 V). To quantify total protein prior to blocking, membranes were stained using Ponceau S staining solution (#A40000279, ThermoFisher, Illkirch-Graffenstaden, France) and imaged using the Amersham Imager 600. The membrane was then immediately blocked in 5% milk Tris-Buffered Saline Tween (TBST) (Tris 20 mM, NaCl 150 mM, Tween 20 0.1% (*w*/*v*)) for one hour. It was then washed thrice in roughly 15 mL TBST under gentle agitation for 5 min each time before being incubated in a sealed sachet with 1/3000 diluted anti-LC3A/B antibody (LC3A/B (D3U4C) XP^®^ Rabbit mAb #12741, Cell signaling, Leiden, Netherlands) or 1/1000 diluted anti-p62/SQSTM1 (SQSTM1/p62 Antibody #5114, Cell signaling, Leiden, Netherlands) in 5% BSA TBST overnight under gentle agitation at 4 °C. The membrane was then thrice washed in TBST for 5 min each time, followed by incubation with roughly 30 mL 1/20,000 diluted anti-rabbit secondary (Peroxidase AffiniPure Goat Anti-Rabbit IgG (H+L), #111-035-144, Jackson) in 5% milk TBST under gentle agitation for 2 h at RT. The membrane was then thrice washed in TBST as before, with membrane signal revealed by the addition of 1 mL ECL reagent (Clarity™ Western ECL Substrate, #1705060S, Bio-Rad, Marnes-la-Coquette, France) to the membrane placed between two transparent plastic sheets. Following incubation for 5 min protected from light, the membrane was then visualised using the Amersham Imager 600. For actin normalisation of loaded protein quantity, the membrane was washed thrice in TBST and then incubated for 2 h with 1/10,000 diluted anti-β-actin (#A5441, Sigma-Aldrich, Saint-Quentin-Fallavier, France) antibody in 5% milk TBST under gentle agitation. The membrane was then washed thrice as before, and incubated with 1/200,000 anti-mouse secondary (Peroxidase AffiniPure Goat Anti-Mouse IgG + IgM (H+L), #115-035-068, Jackson Immunoresearch, Cambridgeshire, United Kingdom), which was incubated, washed and revealed in the same manner as for the anti-LC3 A/B antibody. The LC3-II/LC3-I ratio (a measure of autophagosomal accumulation) was calculated by measurement of integrated density using ImageJ. P62 intensity was measured by integrated density and normalised to total protein content measured by the Ponceau stain (quantified by integrated density).

### 2.10. Lysotracker Green Flow Cytometry

In order to assess the accumulation of acidic lysosomal vesicles within the cells, LysoTracker™ Green DND-26 (#L7526, ThermoFisher, Illkirch-Graffenstaden, France) staining was carried out and measured by flow cytometry. Cells were treated at the desired concentration and duration in 96-well plates before transfer to a V-bottomed 96-well plate (#651101, Greiner Bio-One). The plate was centrifuged (5 min at 350 g, 4 °C), with the supernatant removed by inversion of the plate. The cells were then resuspended in 100 µL Accutase, with the replicates pooled, and left for 5 min at RT to dissociate the spheres. The plate was then centrifuged (5 min at 350 g, 4 °C) with the supernatant eliminated. The cells were resuspended in 50 nM Lysotracker Green diluted in PBS and incubated at room temperature for 15 min protected from light. The cells were then centrifuged (350 g, 5 min, 4 °C) and resuspended in 1/200 diluted PI as a live/dead marker. Cells were run immediately on a FACS Calibur, with the Lysotracker Green analysed in the FL1 channel and PI in the FL3 channel. Live (PI−) cells were counted with dead (PI+) cells excluded from the analysis. Histograms were analysed via FlowJo v10 software.

### 2.11. CD133 Expression

In order to assess whether the “stemness” state of the CSCs could be affected by chemical treatment, CD133 cell surface marker (highly expressed on GSCs) expression was measured via flow cytometry. Dissociated NCH421K cells were treated with the desired concentration(s) of compound (a low/non-toxic dose) by seeding the cells at 7.5 × 10^4^ cells/mL in a 6-well plate (#657160, Greiner Bio-One, Les Ulis, France) (3 mL per well) with the compound. The cells were then incubated for four days under standard culture conditions before recovery of the cells, washing once in PBS and repeated flushing in order to dissociate the spheres without enzymatic treatment which could affect the cell surface marker. The cells were then counted with Trypan Blue (#T8154-100ML, Sigma-Aldrich, Saint-Quentin-Fallavier, France) exclusion for viability analysis using the Countess II FL automated cell counter, with 100,000 cells then centrifuged (350 g, 5 min, 4 °C) and resuspended in 100 µL 1/20 diluted anti-CD133-APC (#17-1338-41, Thermo Fisher Scientific, Illkirch-Graffenstaden, France, Villebon-sur-Yvette, France) antibody or the corresponding isotype control (#400119, Biolegend, Amsterdam, Netherlands) in PBS (2% FBS). The cells were incubated for 1 h on ice protected from light. The cells were centrifuged (350 g, 5 min, 4 °C) and resuspended in 200 µL PBS (2% FBS). The cells were run immediately on the FACS Calibur, with CD133 fluorescence analysed in the FL4 channel. Fluorescence histograms were analysed using FlowJo v10 software.

### 2.12. NCH421K-RAW 264.7 Macrophage Immunogenic Cell Death Co-Culture

In order to assess whether the cell death induced by a compound in GSCs was capable of initiating an immune response, a co-culture assay of treated NCH421K cells with murine macrophage RAW 264.7 cells was carried out. The NCH421K cells were seeded as described in Section 2.2.3. in a 48-well plate (#677180, Greiner Bio-One, Les Ulis, France) format. Spheres were treated at the desired time point and concentration in 300 µL culture volume.

To easily distinguish the RAW 264.7 cells in flow cytometry, they were stained with the stable non-toxic cell membrane marker, CellBrite^®^ NIR680 (#30070, Biotium, Fremont, CA, USA) by incubation with a dilution of 1/2000 in their culture medium at a concentration of 1 × 10^6^ cells/mL for 45 min at 37 °C protected from light. The cells were then washed twice in PBS (200 g, 5 min, RT) before resuspension in RAW cell medium (2% FBS, as higher concentrations of serum disturbed the CSCs) at a concentration of 2 × 10^5^ cells/mL.

In order to avoid toxicity against the immune cells, the plate was centrifuged (350 g, 5 min, RT), with the supernatant carefully aspirated (and retained for DAMPs analysis) in order to avoid disturbing the treated spheroids before replacement with 600 µL stained RAW cell suspension. The co-culture was then left overnight to allow for activation of the immune cells, with cells/supernatants then analysed for activation markers by flow cytometry.

For the sonicated control, to imitate accidental cell necrosis/DAMPs release, wells containing non-treated NCH421K cells were collected by flushing and aspiration of the CSC medium containing cells. The cells were pelleted (350 g, 5 min, RT) and resuspended in RAW cell medium (2% FBS), which was transferred to a glass test tube and sonicated on ice three times at 20 kHz for 10 s on pulsed mode with 30 s intervals using a Vibracell sonicator. A 600 µL stained RAW cell suspension was pelleted (200 g, 5 min, RT), with the supernatant eliminated and the cells resuspended in the sonicated lysate and placed in the original well of the NCH421K cells.

### 2.13. Phagocytosis Assay

In order to assess whether the cell death induced by a compound in GSCs was capable of inducing phagocytosis of these cells by macrophages (and thus indirectly showing the exposure of immune “eat-me” signals), a co-culture assay was carried out as described in Section 2.12. However, in order to track the phagocytosis of the CSCs by the macrophages, the NCH421K cells were also stained using a non-toxic fluorescent marker. Thus, prior to seeding (as it was unsure whether staining of spheroids would produce homogenous staining of the cells), the NCH421K cells were stained with CellTracker™ CM-DiI Dye (#C7000, Invitrogen, Villebon-Sur-Yvette, France) by incubation with a dilution of 1/1000 in their culture medium at a concentration of 5.25 × 10^5^ cells/mL for 5 min at 37 °C protected from light, followed immediately by a further 15 min at 4 °C. The cells were then washed once in PBS and resuspended in their culture medium for seeding at 7.5 × 10^4^ cells/mL in a 48-well plate with 300 µL total volume and left to form spheres for four days. Treatment and co-culture were then carried out as described in Section 2.12. Cells were fixed in 4% paraformaldehyde (PFA) (#47608-1L-F, Sigma-Aldrich, Saint-Quentin-Fallavier, France) PBS for 30 min at RT, centrifuged (350 g, 5 min, RT), resuspended in 200 µL PBS and stored at 4 °C until running on the FACS Canto (Becton Dickinson, Franklin Lakes, New Jersey, United States). NCH421K cells were tracked in the PE channel and RAW 264.7 cells in the APC-Cy7 channel. Fluorescence histograms were analysed using FlowJo v10 software.

### 2.14. ATP Release Assay

In order to assess whether the cell death induced by a compound in GSCs was capable of releasing ATP, an important DAMP in immune cell activation, a luminescence-based ATP assay was carried out in parallel on the supernatant (released ATP) and supernatant + lysed cells (total ATP) of the treated cells. NCH421K cells were treated at the desired time point and concentration in a 48-well plate format (300 µL culture volume), with the supernatant, recovered and centrifuged (500 g, 5 min, RT) in order to remove any cells and cell debris. The supernatant was decanted to a new 1.5 mL Eppendorf containing 300 µL CellTiter-Glo^®^ 3D Cell Viability Assay reagent (Promega, Charbonnières-les-Bains, France) which was mixed by pipetting. At the same time, 300 µL of the same reagent was added to wells containing cells which had been treated in the same manner as those from which supernatant was recovered. The solutions were removed to an Eppendorf and were vigorously mixed to ensure efficient cell lysis. Solutions (including the medium-only control) were incubated for 30 min at RT protected from light. A total of 200 µL was then removed to an opaque 96-well plate in duplicate, with measurement of luminescence emission using a plate reader (SP200 Safas, Monaco). Background luminescence of the culture medium without cells was subtracted, with values expressed as arbitrary RLU counted by the luminometer. The luminescence of the treated supernatant was compared with that of the whole well luminescence to measure the total quantity of cellular ATP in the supernatant.

### 2.15. MHC-II Expression

As another method of assessing immune cell activation from the ICD model described in Section 2.12, RAW 264.7 cells were analysed for their expression of MHC-II. Cells were fixed in 4% PFA PBS for 30 min at RT, centrifuged (350 g, 5 min, RT) and resuspended in 50 µL PBS (2% FBS) with 1/200 diluted MHC-II (#116418, Biolegend, San Diego, CA, USA) or the corresponding isotype controls (#17-4321-81, Thermo Fisher Scientific, Illkirch-Graffenstaden, France) for 30 min on ice. The cells were then washed once in PBS (2% FBS) and ran immediately on the FACSCanto. MHC-II was analysed in the APC fluorescence channel, while RAW 264.7 cells were distinguished and selected in the co-culture through their cell membrane marker in the APC-Cy7 channel. Fluorescence histograms were analysed using FlowJo v10 software.

## 3. Results and Discussion

### 3.1. NHC-Pt(II)-PEI and PEI Decrease CSC Viability and Spheroid Formation

**Figure 1:**
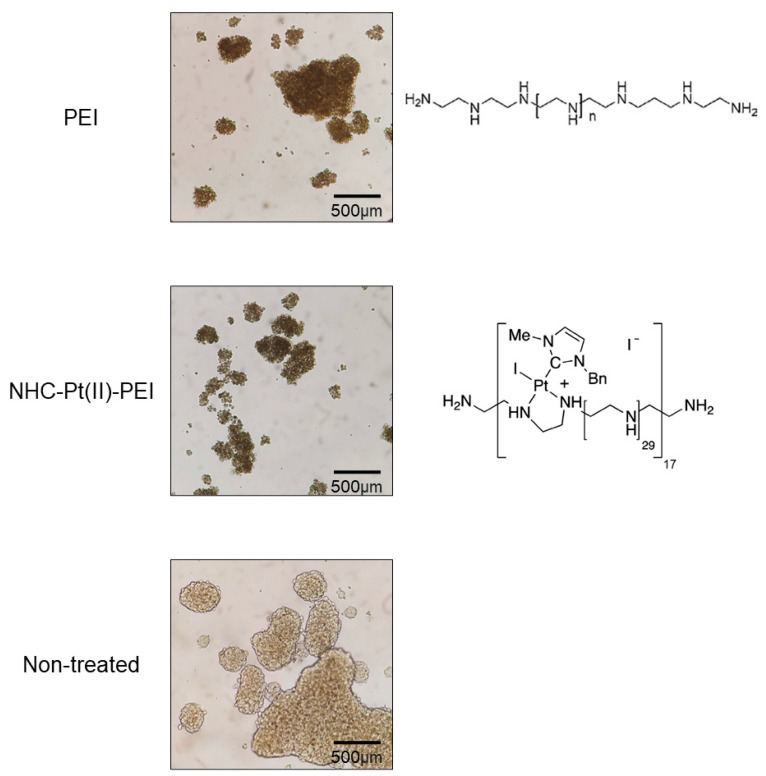
Light microscopy of 24 h treated PEI and NHC-Pt(II)-PEI treated NCH421K spheroids. Inverted light microscopy images at t = 24 h of 2.5 µM Platinum (or equivalent naked PEI) treated NCH421K cells at 4 × 10× = 40× magnification were taken using an Axio Vert A1 inverted light microscope (Zeiss) microscope coupled to a ProgRes C5 cool (Jenoptik, Jena, Germany) camera.

In order to assess the impact of the PDC NHC-Pt(II)-PEI (see structure in Figure 1) and the linear 22 kDa PEI (noted as PEI throughout the manuscript) on CSCs, three GSC cell lines, NCH421K, NCH644 and 3731 were used. These cell lines were isolated from patients suffering from glioblastoma [41,42,44], with their stem-like state being maintained and selected for by growth in a serum-free “stem cell” medium. These cells grow in a naturally non-adherent spheroid morphology (Figure 1). The effect on viability was measured (four days following seeding to allow the formation of spheroids) via ATP decrease using the CelltiterGlo 3D cell viability assay following 24 h treatment and was compared to a more differentiated glioma cell line (U87-MG) and a primary non-cancerous stem cell culture, DPSCs (grown as spheres (Figure S1)) (Table 1 and Figures S2–S6). The commercial platinum-based anti-cancer therapeutic oxaliplatin, and the standard glioblastoma treatment, temozolomide, were used for comparison (previously published results in [45]).

In order to compare concentrations of PDC (which were expressed as the concentration of Pt, of which there are an average of 17 per polymer molecule) with that of its polymer carrier alone (PEI), the true concentration of polymer was multiplied by the average number of platinum atoms which are bound to the same polymer molecule in the PDC, giving concentrations expressed as PEI equivalent (PEI eq). The result was low micromolar IC50 values for both the PDC NHC-Pt(II)-PEI and the "naked" PEI eq against the three GSC lines, significantly lower than the IC50 values against the non-cancerous stem and more differentiated glioma controls. Interestingly, the PEI polymer itself also displayed a high level of toxicity against the GSCs, with a three to tenfold greater toxicity compared to that against the two non-CSC cultures (Table 1). The morphological effect on the spheres of both the PDC and PEI was a drastic darkening, which was accompanied by a potential shrinking, but interestingly without a major disaggregation of the spheroids after 24 h (Figure 1).

This surprising result suggested that the GSCs were specifically sensitive to PEI toxicity, which has been reported once in the literature recently by Prabhakar et al. (2021) [46], who observed that PEI-coated silica nanoparticles presented a high level of toxicity which was seemingly selective to GSCs. A recent study by Knauer et al. (2022) [47] also suggests greater toxicity of a dendrimeric cationic polymer towards GSCs when compared to U87 cells. This is of interest as the use of such polymers as carriers for drugs (as in our case) at concentrations to which non-CSCs are not sensitive, could provide a two-pronged approach for delivering drugs which are known to be effective against bulk tumour cells, while also having a carrier-dependent or linked effect against a polymer-sensitive CSC niche. The chemical conjugation of components to the polymer may also change its physicochemical characteristics favourably by masking a portion of its positive charges, reducing interaction with serum proteins, and thus facilitating its application in vivo [48,49,50], an effect which could be proposed for our PDC from its successful application in vivo without visible side effects on the mice [11]. The PDC holds further promise in this respect as it has been shown to form nanoparticles in solution [11], which may allow its passive accumulation via the EPR effect, physically targeting it to the location of the polymer-sensitive CSCs [12,13].

**Figure 2:**
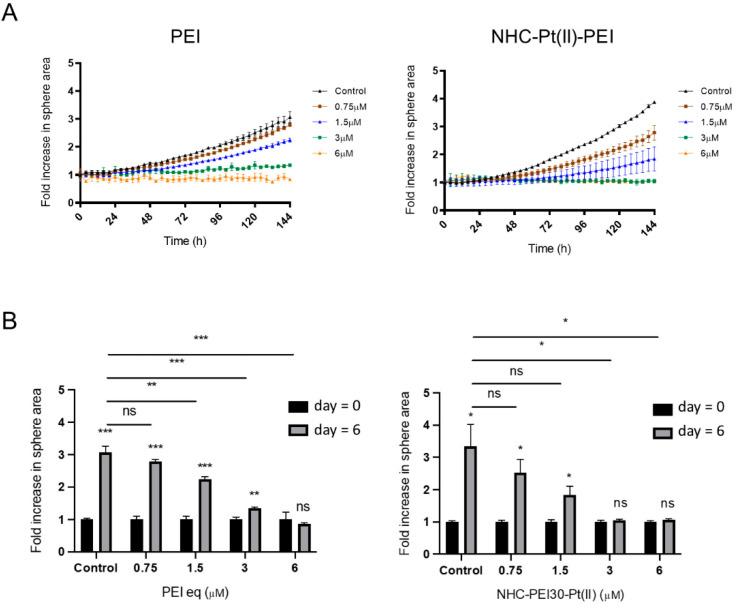
Effect of PEI and NHC-Pt(II)-PEI on NCH421K spheroid formation. (**A**) Scatter plots representing fold increase in spheroid area over time normalised to the time = 0 baseline. (**B**) Paired histograms showing the fold increase in area at time = 0 and time = 6 days (144 h) for each treated condition. Values represent the mean of at least *n* = 3 independent replicates ± one SEM. Control = ethanol vehicle control of the most concentrated condition (6 µM). Statistics above the bar charts represent Student’s *t*-tests carried out between time = 0 and time = day 6 values for each condition. Statistics between histograms represent Student’s *t*-tests of day = 6 values between conditions. ns = *p* > 0.05 * = *p* ≤ 0.05, ** = *p* ≤ 0.01. *** = *p* ≤ 0.001. Distribution normality was confirmed using a Shapiro–Wilk test. Data collected using the IncuCyte and analysed using IncuCyte Zoom software.

The effect of the compounds on the capacity of the GSCs to form spheroids (an in vitro measurement of tumour proliferation capacity) was also investigated by seeding of the cells with treatment and video tracking of the formation of spheres using Incucyte technology (Figure 2).

The result was significant retardation in the ability of the GSCs to form spheres for both the PDC and PEI, showing the compounds may also be capable of interfering with the tumour growth capacity of these cells. 

### 3.2. Culture Media Impacts Polymer Toxicity

**Figure 3:**
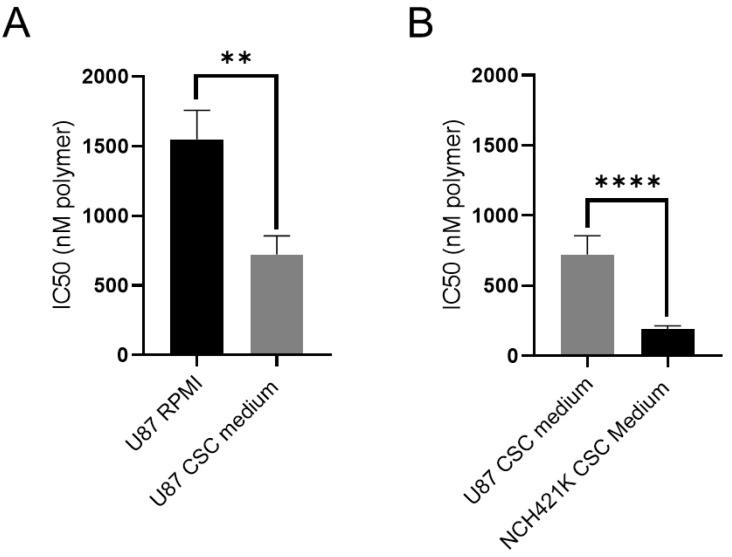
Effect of CSC medium on PEI toxicity on U87-MG cells. (**A**) Histograms showing 24 h IC50 values of PEI (expressed in nM of polymer) on U87-MG cells cultured in their standard medium (RPMI 10% FBS) vs. in serum-free CSC medium. (**B**) Histograms showing 24 h IC50 values of PEI (expressed in nM of polymer) on U87-MG cells cultured in CSC medium vs. NCH421K GSCs cultured in CSC medium. Values represent the mean of at least *n* = 3 independent experiments ± one SEM. Statistics represent Student’s T-tests. ** = *p* ≤ 0.01, **** = *p* ≤ 0.0001. Distribution normality was confirmed using a Shapiro–Wilk test.

As the in vitro model we used was limited with respect to the physiologically real environment and behaviour of GSCs, we particularly wished to investigate whether the serum-free nature of the CSC medium would have an impact on the physicochemical environment of the PEI such that it could change its toxicity, and potentially be responsible for the observed higher toxicity of the polymer towards CSCs. We thus treated the more differentiated glioma U87 cells for 24 h with the polymer in their classic 10% FBS medium and compared it with the treatment when applied in the FBS-free CSC medium (Figure 3). 

The result showed there was indeed a significant reduction of PEI’s IC50 against U87 cells in the CSC medium (a roughly 800 nM difference), indicating that such difference in conditions may indeed influence the measurement of the cytotoxicity of compounds. This is an important observation for in vitro CSC studies, showing that great care should be taken in comparing toxicity between cell lines cultured in greatly different media. However, despite the impact on cytotoxicity, even when accounting for the difference in cellular medium composition, a roughly four-fold difference in IC50 remained between the GSCs and non-stem glioma (Figure 3B). This suggested that a specific sensitivity of the GSCs towards the polymer did indeed exist. 

### 3.3. NHC-Pt(II)-PEI and PEI Induce Rapid Membrane Permeabilisation and Cytoplasm Vacuolisation in GSCs

**Figure 4:**
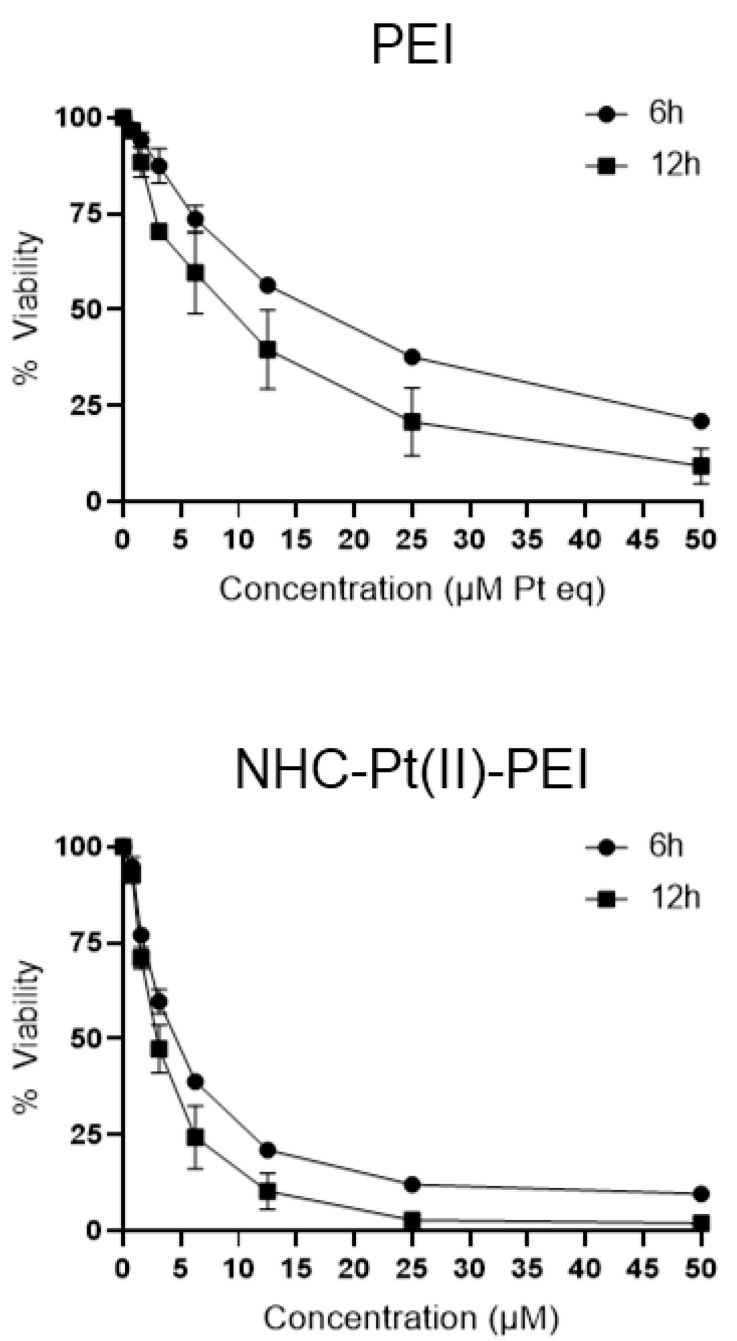
PEI and NHC-Pt(II)-PEI cell death kinetics. Cell viability curves of 6 h and 12 h PEI and NHC-Pt(II)-PEI treated NCH421K spheroids were measured via the CelltitreGlo 3D cell viability assay. Values are the mean of *n* = 3 independent replicates ± one SEM.

The next step of the study was to elucidate the mechanism of cell death induced by PEI or NHC-Pt(II)-PEI. For this, an investigation into the kinetics of the GSC spheroid viability reduction of the compounds showed a rapid mechanism of action with major toxicity occurring after only 6 h of treatment, with the PDC seemingly inducing toxicity more quickly than PEI alone (Figure 4).

Such rapid toxicity is consistent with previous studies into the mechanisms of PEI toxicity [51], while the somewhat greater and faster toxicity of the PDC may be due to its physicochemical change into nanoparticles, or due to an implication of the platinum’s chemistry.

**Figure 5:**
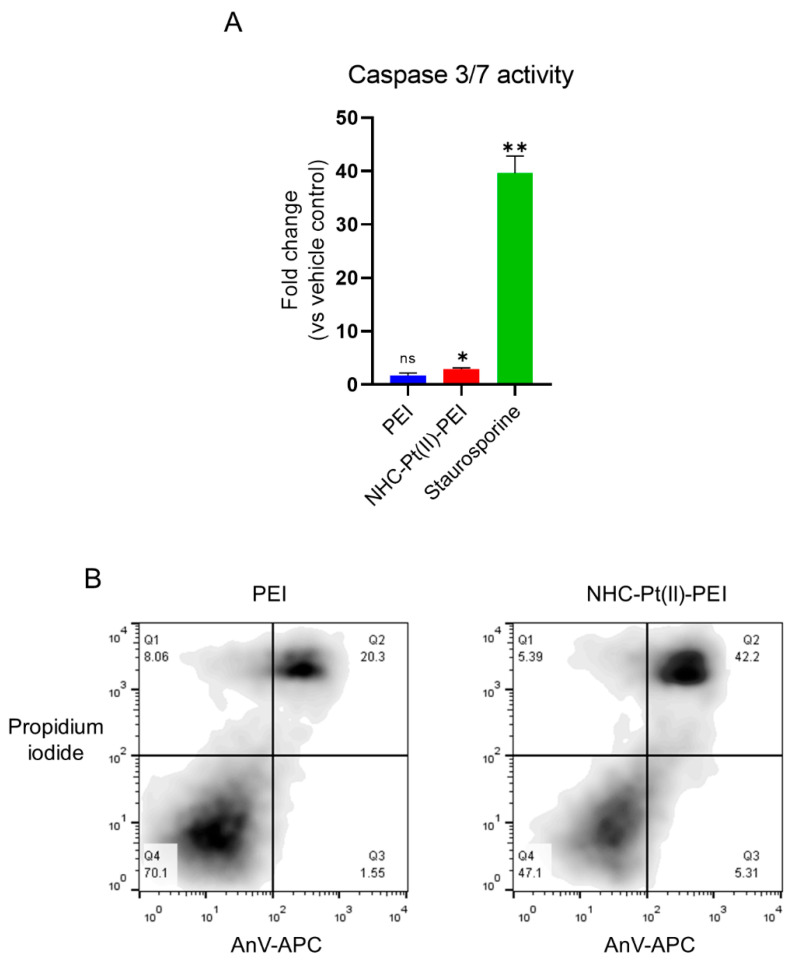
Absence of apoptotic markers on 24 h 2.5 µM NHC-Pt(II)-PEI and 2.5 µM PEI eq treated NCH421K spheroids. (**A**) Histograms showing fold change luminescence intensity compared to the corresponding vehicle control. Values represent the mean of at least *n* = 3 independent experiments ± one SEM. Statistics represent Student’s t-tests with a Welch’s correction vs. the corresponding vehicle reference. Distribution normality was confirmed using a Shapiro–Wilk test. ns = *p* > 0.05, * = *p* ≤ 0.05, ** = *p* ≤ 0.01. (**B**) Flow cytometry dot plots (FlowJo v10) of treated NCH421K cells stained with AnV-APC and PI. Viable cells = AnV−/PI−. Early apoptotic cells = AnV+/PI−. Late apoptotic/necrotic cells = AnV+/PI+. Early necrotic cells = AnV−/PI+.

We also wished to investigate whether the cell death mechanism induced was apoptotic, as activation of apoptotic markers has been identified in other studies and suggested as a dominant driver of cell death [52,53,54]. This was carried out by the measurement of caspase 3/7 activity (the terminal executioner caspases of apoptosis) and by flow cytometric measurement of early phosphatidylserine exposure on the outer cell membrane of cells which still maintain their membrane integrity AnV+/PI− cells). The result showed a clear absence of early phosphatidylserine externalisation as well as a lack of major caspase 3/7 activation for both the PDC and PEI (Figure 5A,B). This is contrary to the phosphatidylserine externalisation shown for the PDC in a previous study against the HCT116 colorectal cancer cell line [11].

This suggested that apoptosis was not the driver of cell death for PEI and the PDC against the GSCs, which is supported by another study where apoptotic markers were not identified and the caspase inhibitor zVAD-fmk had no effect on PEI-induced cell death [51]. We confirmed this for the PDC used in this study (Figure S7).

**Figure 6:**
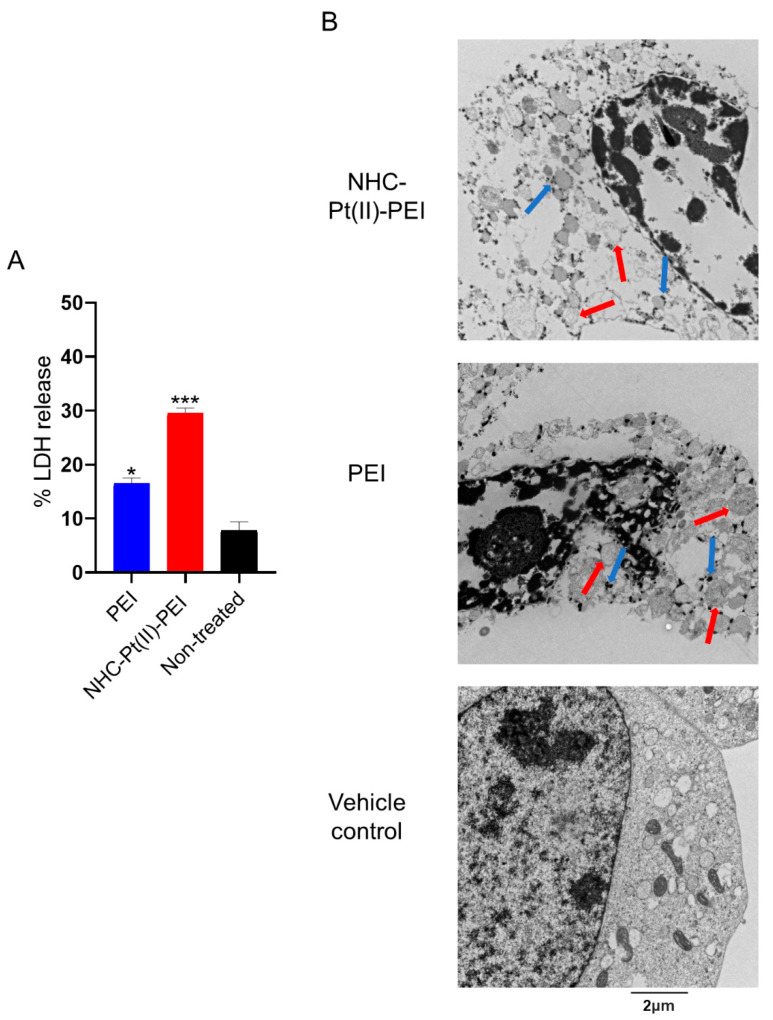
LDH release and necrotic, vacuolised cell death morphology of 6 h 5 µM NHC-Pt(II)-PEI and 5µM PEI eq treated NCH421K spheroids. (**A**) Histograms showing quantified LDH activity of cell supernatant as a percentage of the activity of lysed non-treated cells. Values represent the mean of at least *n* = 3 independent experiments ± one SEM. Statistics represent Student’s *t*-tests versus the non-treated condition. Distribution normality was confirmed using a Shapiro–Wilk test. * = *p* ≤ 0.05, *** = *p* ≤ 0.001. (**B**) Representative TEM images of the treated cells at 15,000× magnification. Red arrows = Double membraned vesicles. Blue arrows = Purported lysosomes.

As other studies have shown PEI is able to induce a rapid perturbation of cell membrane integrity, resulting in necrosis [51,53], we thus wished to confirm this by treatment of NCH421K spheroids for 6 h with the compounds. This was followed by quantification of the release of LDH (an intracellular metabolic enzyme) by measurement of its enzymatic activity in the cellular supernatant and observation of cell death morphology by TEM. The result showed a significant release of total cellular LDH following 6 h (Figure 6A).

This membrane permeabilisation was confirmed through electron microscopy (Figure 6B), which also revealed a significant nuclear condensation and vacuolisation of the cytoplasm (Figure 6B), which was consistent with an increased granularity observed on the forward-scatter (FSC)/side-scatter (SSC) dot plots on the flow cytometer (Figure S8). This thus confirmed that the cell death (against GSCs) of PEI and the PDC proceeds via rapid membrane permeabilisation and a highly vacuolised necrosis-like cell death. 

### 3.4. NHC-Pt(II)-PEI and PEI Induce a Protective Autophagy Response

Amongst the numerous cytoplasmic vesicles, some double-membraned vesicles could be identified, which indicated an accumulation of autophagosomes, as well as smaller dark vesicles which were likely to be lysosomes [55]. This led to the hypothesis that the observed vacuolised morphology was linked to an implication of the autophagy-lysosomal pathway, which is an important cellular turnover/recycling mechanism [26]. Either through a high level of activation of the autophagic pathway or through inhibition of autophagosome-lysosome fusion, causing their accumulation. Autophagy has been shown to be upregulated and used as a survival and drug resistance mechanism in CSCs which is highly implicated in the maintenance of their “stemness” [25,56], with therapeutics targeting this pathway, thus being of greater interest [57,58].

**Figure 7:**
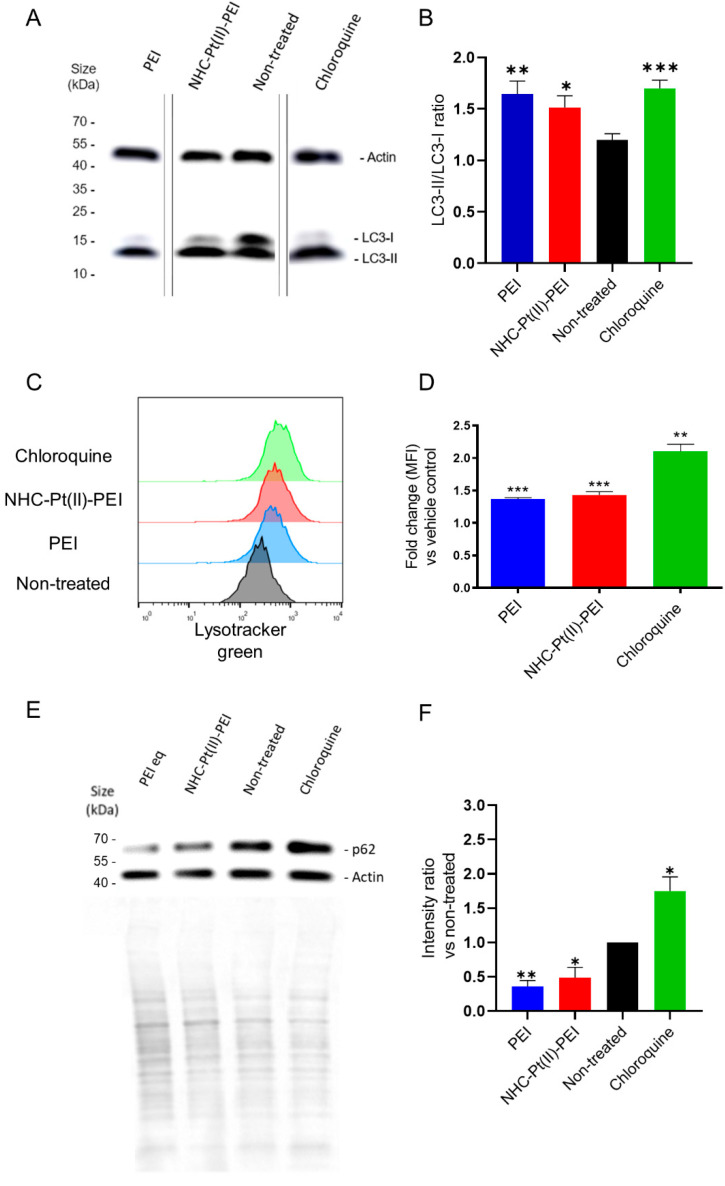
Accumulation of LC3-II and acidic vesicles accompanied by increased p62 degradation in 6 h 5 µM NHC-Pt(II)-PEI, 5 µM PEI eq treated NCH421K spheroids. (**A**) Representative Western blot of total cell lysate showing accumulation of LC3-II (lipid-bound) and reduction of free LC3-I. (**B**) Histograms showing LC3-II/LC3-I intensity ratio calculated from the integrated density of selected bands using ImageJ. Values represent the mean of at least *n* = 3 independent experiments ± one SEM. Statistics represent Student’s *t*-tests vs. the non-treated control. Distribution normality was confirmed using a Shapiro–Wilk test. (**C**) Representative fluorescence histograms (FlowJo v10) showing lysotracker green fluorescence of live gated NCH421K cells. (**D**) Histograms showing fold change in median lysotracker fluorescence vs. control of the treated conditions. Values represent the mean of at least *n* = 3 independent experiments ± one SEM. Statistics represent Student’s T-tests with a Welch’s correction vs. the corresponding vehicle reference. Distribution normality was confirmed using a Shapiro–Wilk test. * = *p* ≤ 0.05, ** = *p* ≤ 0.01, *** = *p* ≤ 0.001. MFI = median fluorescence intensity. (**E**) Representative Western blot of total cell lysate showing changes in p62 levels. Below shows the corresponding actin and Ponceau S total protein loading controls. (**F**) Histograms showing the ratio of p62 expression (calculated from the integrated density of selected bands using Image J) versus the non-treated control, normalised for total protein loading differences by calculated Ponceau S lane intensities. Values represent the mean of *n* = 4 independent experiments + one SEM. Statistics represent Student’s *t*-tests. Distribution normality was confirmed using a Shapiro–Wilk test. * = *p* ≤ 0.05, ** = *p* ≤ 0.01.

In order to verify whether treatment with the PDC and PEI induce an accumulation of autophagosomal vesicles, the NCH421K cells were treated for 6 h. Cells were then lysed, with total cellular lysate then analysed via SDS-PAGE Western blot for an accumulation of the protein LC3-II (lipidated LC3). LC3 is an important protein involved in autophagy and autophagosomal formation which inserts itself into the autophagosomal membrane following lipidation with the phospholipid, phosphatidylethanolamine (PE). Thus, an accumulation of autophagosomal vesicles may be detected by the differential migration of lipidated vs. non-lipidated (LC3-I) LC3. Such an accumulation may be due either to an increase in autophagic flux, and thus an increased activation of autophagy, or due to an inhibited turnover of autophagosomes, with turnover implicating the de-lipidation and degradation of LC3-II [59]. Inhibition of LC3-II turnover may be caused by an inhibition of lysosome-autophagosome fusion (the autophagolysosome), as the final degradation and turnover of the sequestered contents are dependent on this step [27,59]. The known inhibitor of lysosome-autophagosome fusion, chloroquine [60,61] was thus used as a positive control for LC3-II accumulation [59]. Treatment with the PDC and PEI showed a significant increase in the LC3-II/LC3-I ratio, indicative of autophagosome accumulation (Figure 7A,B and Figure S11).

In order to confirm whether there was also a corresponding accumulation of acidic vesicles in the cells, the treated cells were stained with the lysosomotropic dye (acidic vesicle accumulating) Lysotracker Green. Chloroquine again served as a positive control, which has been shown to increase cellular lysosome volume [60,61,62,63]. The compounds showed a clear increase in the lysotracker signal (Figure 7C,D). This suggests an accumulation of lysosomes for the PDC and PEI, as for chloroquine. However, the cellular morphology of chloroquine-treated cells observed by electron microscopy did not show the same drastic vacuolisation (Figure S9). One might suspect that the described “proton-sponge” effect of PEI [64,65] may lead to lysosomal dysfunction which prevents fusion with autophagosomes in a manner similar to chloroquine. However, a similar LC3-II accumulation we observed for poly-L-lysine (PLL)-treated cells (Figure S10), a cationic polymer whose pKa does not allow the proton-sponge effect to occur, suggested this to not be the case.

To confirm this, the treated cells were blotted for p62, a ubiquitin-binding protein used for the targeting of proteins for selective autophagy which is itself degraded by autophagy, and thus whose levels inversely correlate to autophagic activity [59,66]. The observation of a decrease in p62 degradation shows that the PEI and PDC-induced increase in autophagy-related vesicles is due to increased activation of the pathway, contrary to the autophagic block induced by chloroquine which increases p62 levels (Figure 7E,F and Figure S11). This is in agreement with two other studies which have shown a similar increase in autophagy due to PEI treatment [67,68] as well as another study into the effect of polystyrene nanoparticles known to cause lysosomal damage which also increased autophagic flux [69]. Interestingly, this latter study concluded that autophagy induction at the early stage is an initial pro-survival response to the treatment but also that lysosomal dysfunction eventually leads to inhibition of the pathway later on [69].

**Figure 8:**
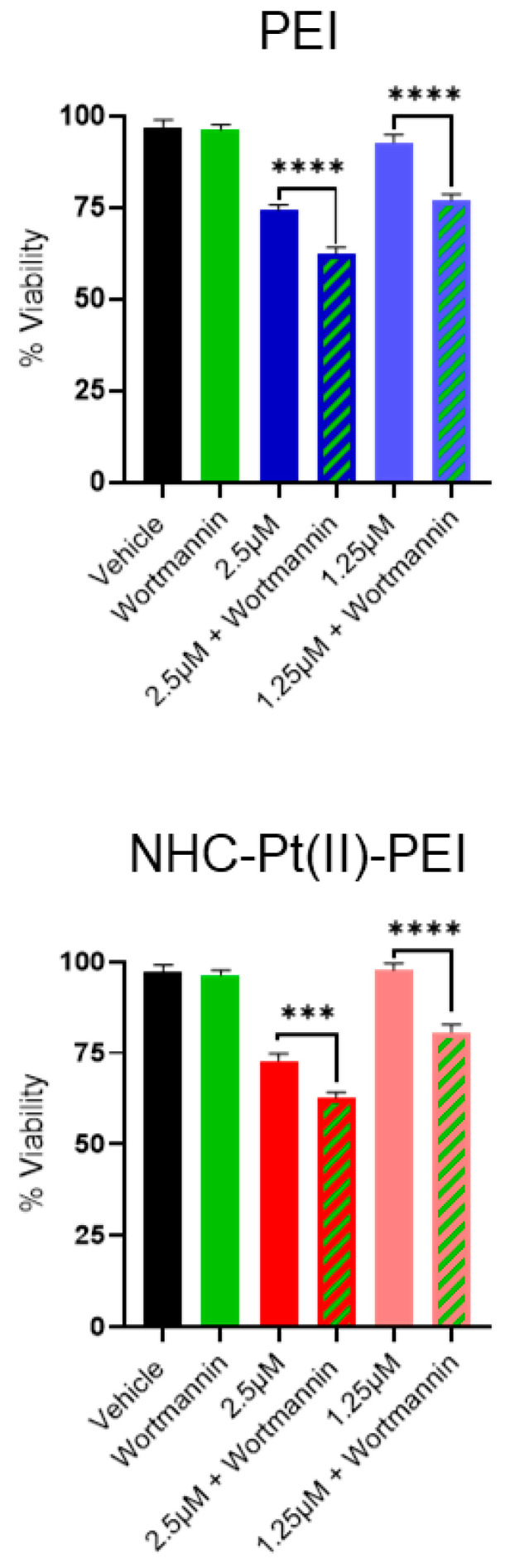
Increased PEI and NHC-Pt(II)-PEI toxicity with wortmannin co-treatment. CelltiterGlo 3D cell viability assay of NCH421K cells treated for 24 h with PEI or NHC-Pt(II)-PEI co-treated with 50 nM wortmannin (following 2 h pre-treatment with 50 nM wortmannin). Vehicle control corresponds to the equivalent highest amount of solvent added. Values represent the mean of at least *n* = 3 independent experiments ± one SEM. Statistics represent Student’s *t*-tests. Distribution normality was confirmed using a Shapiro–Wilk test. *** = *p* ≤ 0.001. **** = *p* ≤ 0.0001.

This role as a protective mechanism is also indicated in our case, as co-treatment with wortmannin (an inhibitor of the critical autophagy regulator phosphoinositide 3-kinase (PI3K)) [70] significantly increased the induced toxicity of treatment with PEI and the PDC (Figure 8).

Autophagy has been shown to be a highly important and finely regulated mechanism in CSCs, which is implicated in the maintenance of their stem-like phenotype [56,71]. Its exact role is somewhat controversial, with studies suggesting that both induction [72,73] and inhibition [74,75] are capable of interfering with the stem-like phenotype (indicating that balance, rather than activation or inhibition, may be the key to its role), which may have major therapeutic implications for their metastasis and drug-resistance [71,76]. We thus wondered whether the evident implication in the autophagic pathway displayed by PEI and our PDC could have an effect on the stem-like phenotype of the cells.

### 3.5. NHC-Pt(II)-PEI and PEI Treatment Reduce the Expression of CSC Marker CD133

**Figure 9:**
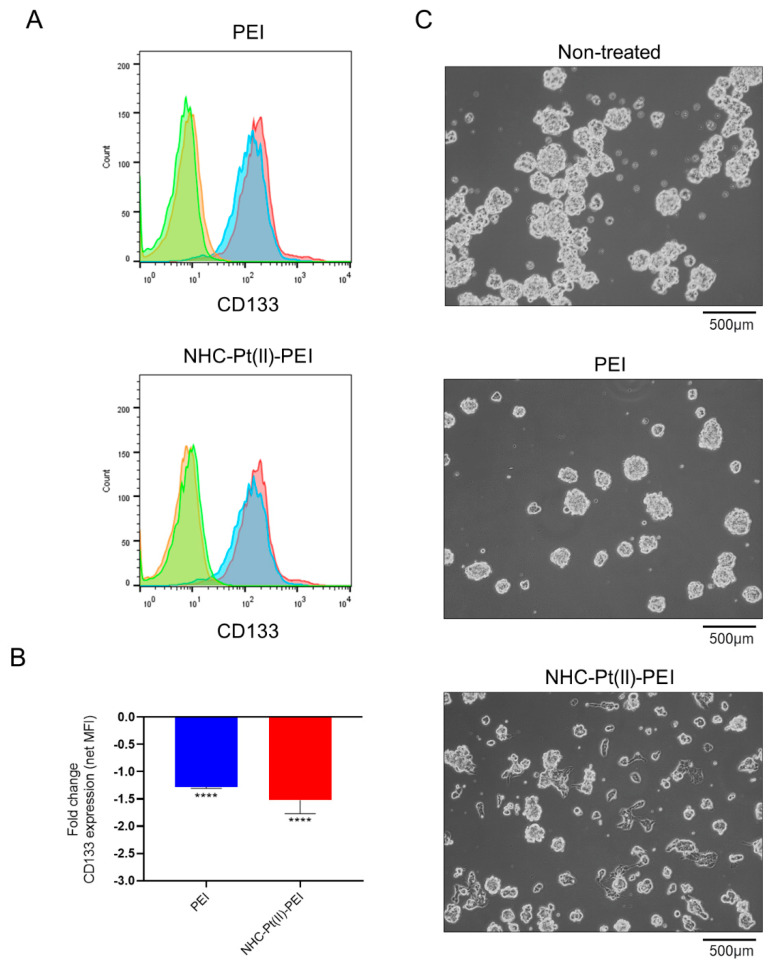
Loss of CD133 CSC marker and CSC morphology of NCH421K cells treated for 4 days with 375 nM NHC-Pt(II)-PEI and PEI eq. (**A**) Representative flow cytometry fluorescence histograms (FlowJo v10) showing a reduction in CD133 fluorescence on NCH421K cells. Orange = EtOH control isotype. Green = PEI or NHC-Pt(II)-PEI treated isotype. Red = Vehicle-treated CD133 stained. Blue = PEI or NHC-Pt(II)-PEI CD133 stained. (**B**) Histograms showing fold change in CD133 net geometric mean fluorescence intensity (MFI stained—MFI isotype) vs. vehicle-treated control. Values represent the mean of at least *n* = 3 independent experiments. Statistics represent Student’s *t*-tests with a Welch’s correction vs. the corresponding vehicle reference. Distribution normality was confirmed using a Shapiro–Wilk test. **** = *p* ≤ 0.0001. (**C**) Inverted light microscopy images of treated NCH421K cells at 4 × 10× = 40× magnification were taken using an Axio Vert A1 inverted light microscope (Zeiss) microscope coupled to a ProgRes C5 cool (Jenoptik, Jena, Germany) camera.

In order to evaluate whether the PDC and PEI could cause differentiation of the NCH421K GSCs, the expression of the GSC marker CD133 was evaluated using flow cytometry following treatment with low concentrations (375 nM and 375 nM PEI eq) of the compounds for four days, in order to avoid significant cell death while giving time for significant cellular expression changes to occur. Both induced a slight but significant reduction in CD133 expression (Figure 9A,B), indicating a reduction in the stem-like phenotype, as U87-MG cells did not express CD133 (Figure S12).

Interestingly, this was also accompanied by a change in morphology from spheroids to a more classical adherent neural morphology for the PDC-treated condition (Figure 9C), which was less evident for the PEI-treated condition but induced an adherence of the spheroids to the culture plate (Figure 9C). This shows the great promise of PEI as a carrier for anti-CSC therapeutics, since the delivery agent itself, as well as potentially having a specific affinity or toxicity towards the CSC population, may sensitise the cells to a cargo towards which they may otherwise be resistant, and which may be naturally effective against the rest of the bulk tumour mass [75].

### 3.6. NHC-Pt(II)-PEI and PEI Cell Death Induces Phagocytosis and DAMPs Release

The observed cell death mode of NHC-Pt(II)-PEI and PEI was of interest in relation to the anti-cancer immune response. This was due to its necrotic nature, which is known to be able to induce an anti-cancer immune response through the release of DAMPs [77], and also due to its activation of autophagy. As autophagy has been shown to be important for tumour immunogenicity via the secretion of the DAMP ATP [78], and the presentation of antigen on tumour cells (although autophagy’s role in the anti-cancer immune response is complex, as it also seems to be important in immune evasion of established tumours) [79].

**Figure 10:**
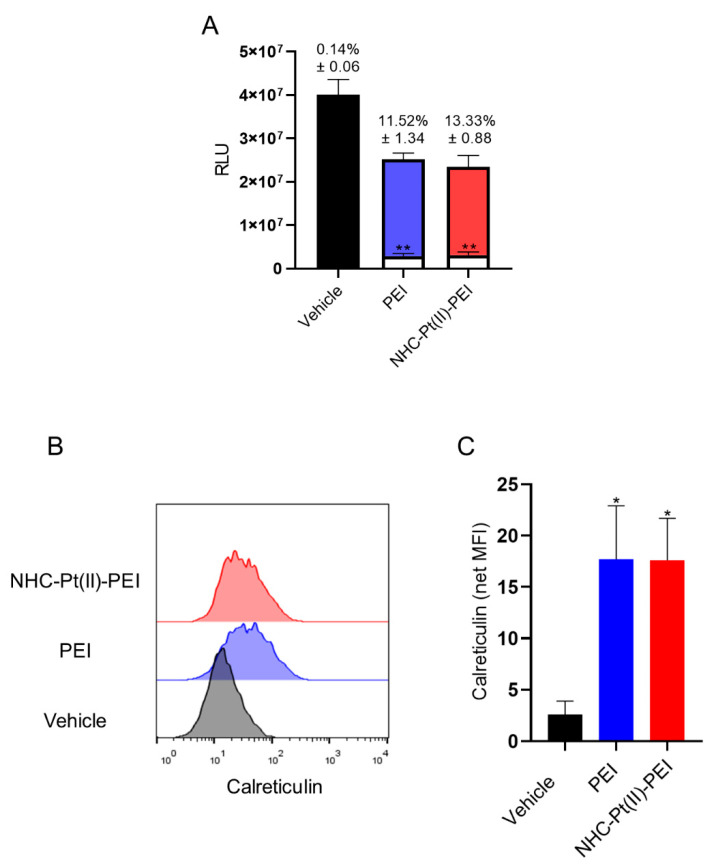
DAMPs release of 6 h 5µM NHC-Pt(II)-PEI and 5µM PEI eq treated NCH421K spheroids. (**A**) Overlapped histograms of ATP generated RLU using the CelltiterGlo 3D viability assay measured on the cellular supernatant (white) and the total well contents (cells + supernatant) (coloured) following treatment. The numbers above represent the percentage of ATP found in the supernatant relative to the total well contents. Values represent the mean of at least *n* = 3 independent experiments ± one SEM. Statistics represent Student’s t-tests vs. the corresponding vehicle reference. Distribution normality was confirmed using a Shapiro–Wilk test. ** = *p* ≤ 0.01. (**B**) Representative flow cytometry histograms (FlowJo v10) of treated live-gated (PI-) cells stained for calreticulin. (**C**) Histograms of net MFI (median) (MFI condition – MFI isotype control) of treated cells. Values represent the mean of at least *n* = 3 independent experiments ± one SEM. Statistics represent Student’s T-tests vs. the corresponding vehicle reference. Distribution normality was confirmed using a Shapiro–Wilk test. * = *p* ≤ 0.05.

To evaluate the potential effect of NHC-Pt(II)-PEI and PEI-induced cell death on anti-tumour immune response induction, a co-culture system was used. NCH421K cells were treated for 6 h with the compound to initiate significant (but not total) cell death, with the treatment then removed from the cells and replaced with a culture of RAW 264.7 murine macrophage cells and left overnight. The removed supernatant was first dosed for a release of ATP (which is detected as a DAMP by purinergic receptors on immune cells) using the CelltiterGlo 3D assay. The result was a significant release of ATP following 6 h treatment, with roughly 12% of total cellular ATP being present in the cell supernatant (Figure 10A).

**Figure 11:**
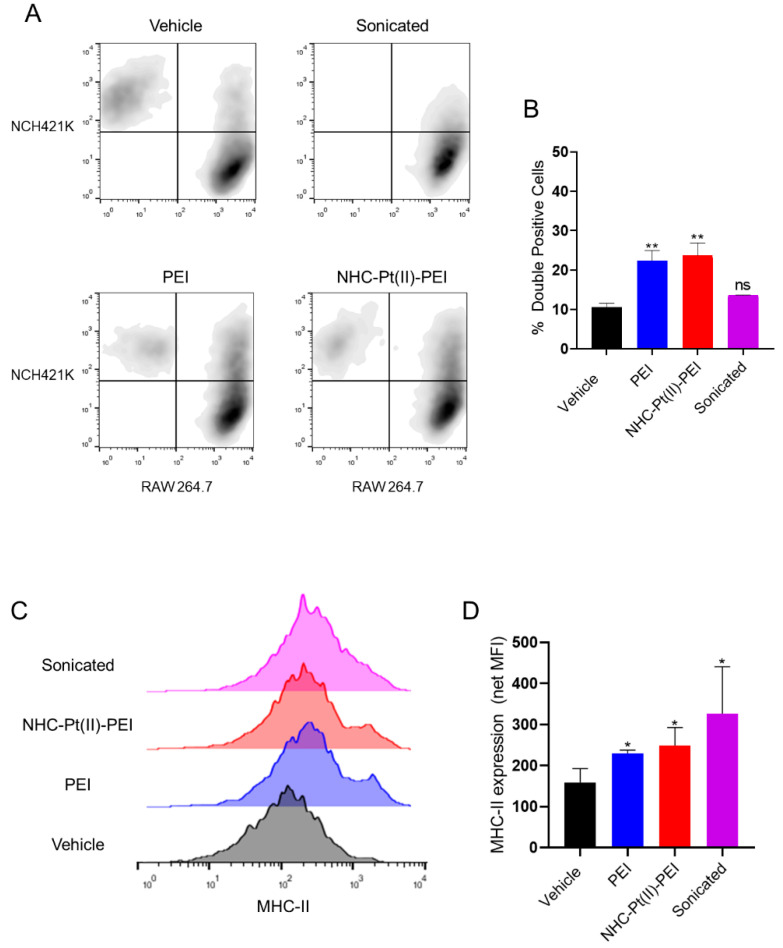
Phagocytosis and macrophage activation by 6 h 5µM NHC-Pt(II)-PEI and 5µM PEI eq treated NCH421K spheroid co-culture. (**A**) Representative flow cytometry dot plots (FlowJo v10) of stained NCH421K spheroids treated for 6 h prior to addition of stained RAW macrophages for overnight co-culturing. (**B**) Histograms showing the percentage of double-positive (RAW phagocytosed NCH421K) cells representing the mean of *n* = 3 independent experiments ± one SEM. Statistics represent Student’s t-tests vs. the corresponding vehicle reference. Distribution normality was confirmed using a Shapiro–Wilk test. ns = *p* > 0.05, ** = *p* ≤ 0.01. (**C**) Representative flow cytometry histograms (FlowJo v10) of MHC-II fluorescence. (**D**) Histograms showing net MFI (median) (MFI treatment—MFI isotype control) of treated cells. Values represent the mean of *n* = 3 independent experiments. Statistics represent Student’s *t*-tests vs. the corresponding vehicle reference. Distribution normality was confirmed using a Shapiro–Wilk test. * = *p* ≤ 0.05.

The externalisation of the phagocytotic DAMP calreticulin from the ER to the cell surface was measured via flow cytometry, which showed an externalisation of the protein to the surface of cells with an intact plasma membrane (Figure 10B,C). Expression of “eat me” signals such as calreticulin is the first step for the phagocytosis of tumour cells. Thus, we followed the active phagocytosis of these cells by RAW mouse macrophages using a flow cytometric phagocytosis assay, where the RAW cell population showed a clear uptake of the NCH421K cells (whose membranes were rendered fluorescent with a stable membrane dye prior to seeding and treatment with the compounds) for the PDC and PEI-treated conditions, but not for the sonicated control (consisting of sonicated NCH421K lysate to imitate “accidental” necrosis/DAMP release), indicating that dye uptake was indeed due to phagocytosis of whole cells and not due to non-specific pinocytosis of free or cellular debris-bound dye (Figure 11A,B).

Another important feature of ICD is the induction of APC maturation by released DAMPs that will permit the initiation of an adaptive immune response. The RAW cells also showed an upregulation of MHC-II, a marker of macrophage maturation, showing that PDC and PEI induced-cell death have the potential to activate the adaptive immune response (Figure 11C,D). This shows that the necrotic cell death type induced by the PDC and PEI against GSCs shows characteristics in line with the ability to induce an immune response, a highly desirable characteristic of anti-cancer chemotherapeutics as the activation of an adaptive immune response against tumours is well known to greatly improve clinical outcomes [36,80].

## 4. Conclusions

This work, originally intended as an evaluation of the previously described anti-cancer platinum PDC NHC-Pt(II)-PEI [11] against the therapeutically important CSC sub-population, may potentially be one of the first of many studies in a new paradigm within the search for effective methods of treating CSCs. The cationic polymer, linear 22 kDa PEI, may present a potent and specific toxicity toward CSCs. This effect seems to be maintained when administered in the form of a PDC, with the physicochemical changes induced by this conjugation being potentially crucial for its pharmaceutical tolerance. The cell death was shown to be necrotic, rather than apoptotic in nature, potentially bypassing the resistance to apoptosis of CSCs and showing promise as an inducer of an anti-cancer immune response. The cell death was also shown to be accompanied by an induction of a protective autophagy response. The implication of the autophagic pathway in the compound’s mechanism of action is a highly promising characteristic in its application against CSCs, as it is a pathway for which balance is key in the maintenance of their metastatic and drug-resistant phenotype. A phenotype in which we observed interference through the reduction of the CSC marker CD133. One could thus envisage the exploitation of such effects on CSCs for the delivery of a chemotherapeutic payload to which CSCs are normally resistant, with either direct toxicity or a sensitisation to the payload occurring through the action of the polymer carrier. Being, so far, one of the very few studies to suggest that CSCs possess a sensitivity to the toxicity of polycations, further attention must be paid to these observations to elaborate on why this may be the case, and to exactly which molecules it may apply. Thus bringing polymers and PDCs to the forefront of the fight against this clinically nefarious niche.

## Figures and Tables

**Table 1:** IC_50_ values (µM) of the compounds after 24 h of treatment.

	Glioblastoma Stem Cells			Non-Cancer Stem Cells	Glioma Cells
	NCH421K	NCH644	3731	DPSC	U87
**Oxaliplatin**	53 ± 1.9	55 ± 1.6	55.9 ± 2	>100	>100
**Temozolomide**	>100	97.2 ± 15	>100	>100	>100
**NHC-Pt(II)-PEI**	2.6 ± 0.1	1.4 ± 0.3	2.1 ± 0.3	6.6 ± 0.9 ****^, ####, xxxx^	9.3 ± 0.8 ****^, ####, xxxx^
**PEI (eq)** ***PEI (nM polymer)***	3.3 ± 0.4 *(194)*	10.6 ± 0.6 *(624)*	2.9 ± 0.2 *(169)*	32.5 ± 0.9 ****^, ####, xxxx^ *(1912)*	26.3 ± 3.5 ****^, ###, xxxx^ *(1547)*

All IC_50_ values are the mean of at least *n* = 3 independent experiments ± one standard error of the mean (SEM), with each experiment carried out in at least a technical duplicate. IC50 values were calculated by non-linear regression of cell viability curves generated via CelltitreGlo 3D cell viability assay using Prism software. Statistics (calculated using Prism) represent one-way ANOVA tests of the three GSC lines with either DPSCs or U87 cells with a post hoc Dunnett’s multiple comparisons test against DPSC or U87 values as control. * = DPSC/U87 vs. NCH421K, ^#^ = DPSC/U87 vs. NCH644 and ^x^ = DPSC/U87 vs. 3731. ^###^ = *p* ≤ 0.001; ****/^####^/^xxxx^ = *p* ≤ 0.0001. Distribution normality was confirmed using a Shapiro–Wilk test. NHC-Pt(II)-PEI values are expressed as the concentration of Pt.

## Data Availability

Data are contained within the article or Supplementary Material. Raw data may be made available upon request.

## Supplementary Materials

The following supporting information can be downloaded at: https://www.mdpi.com/article/10.3390/cancers14205057/s1, Figure S1. DPSC spheroid cell culture; Figure S2. IC50 for the GSC cell line NCH421K; Figure S3. IC50 for the GSC cell line NCH644; Figure S4. IC50 for the GSC cell line 3731; Figure S5. IC50 for the non-cancerous stem cells, DPSCs; Figure S6. IC50 for the non-stem glioma cell line U87-MG; Figure S7. Cell death proportion of 24 h NHC-Pt(II)-PEI and zVAD-fmk co-treated NCH421K cells; Figure S8. The granularity of 24 h 5 μM PEI eq and NHC-Pt(II)-PEI treated NCH421K spheroids; Figure S9. Transmission electron microscopy of 6 h 60 μM chloroquine treated NCH421K cells; Figure S10. Unedited LC3 western blots; Figure S11. Unedited p62 western blots; Figure S12. U87 CD133 expression.

## Abbreviations

AnV	Annexin V
APC	Allophycocyanin/Antigen-presenting cell
CSC	Cancer stem cell
DAMP	Damage associated molecular pattern
DER	Poly(propylene glycol) diglycidyl ether
DMEM	Dulbecco's Modified Eagle Medium
DMAE	Dimethylethanolamine
DPSC	Dental pulp stem cell
EPR	Enhanced permeability and retention effect
FBS	Fetal bovine serum
FSC	Forward scatter
GSC	Glioblastoma stem cell
ICD	Immunogenic cell death
LC3	protein microtubule-associated proteins 1A/1B light chain 3
LDH	Lactate dehydrogenase
MEM	Minimum essential medium
MFI	Median/Geometric mean fluorescence intensity
NHC	N-heterocyclic carbene
NSA	Nonenyl succinic anhydride
PBS	Dulbecco’s phosphate-buffered saline
PDC	Polymer–drug conjugate
PEG	Polyethylene glycol
PEI	Polyethylenimine
PI	Propidium iodide
RLU	Relative luminescence unit
RPMI	Roswell Park Memorial Institute
SEM	Standard error of the mean
SDS-PAGE	Sodium dodecyl sulphate–polyacrylamide gel electrophoresis
SSC	Side scatter
TEM	Transmission electron microscopy

## References

[1] Dilruba S., Kalayda G.V. (2016). Platinum-Based Drugs: Past, Present and Future. Cancer Chemother. Pharmacol..

[2] Aggarwal M., Chawla S., Singh K., Rana P. (2018). Evaluation of Anticancer Drug Utilization and Monitoring of Adverse Drug Reaction in the Indoor Patients Receiving Cancer Chemotherapy in a Tertiary Care Hospital in New Delhi. J. Basic Clin. Pharm..

[3] Pages B.J., Garbutcheon-Singh K.B., Aldrich-Wright J.R. (2017). Platinum Intercalators of DNA as Anticancer Agents. Eur. J. Inorg. Chem..

[4] Dasari S., Bernard Tchounwou P. (2014). Cisplatin in Cancer Therapy: Molecular Mechanisms of Action. Eur. J. Pharmacol..

[5] Zhang C., Xu C., Gao X., Yao Q. (2022). Platinum-Based Drugs for Cancer Therapy and Anti-Tumor Strategies. Theranostics.

[6] Skander M., Retailleau P., Bourrié B., Schio L., Mailliet P., Marinetti A. (2010). N-Heterocyclic Carbene-Amine Pt(II) Complexes, a New Chemical Space for the Development of Platinum-Based Anticancer Drugs. J. Med. Chem..

[7] Bellemin-Laponnaz S. (2020). N-Heterocyclic Carbene Platinum Complexes: A Big Step Forward for Effective Antitumor Compounds. Eur. J. Inorg. Chem..

[8] Ekladious I., Colson Y.L., Grinstaff M.W. (2019). Polymer–Drug Conjugate Therapeutics: Advances, Insights and Prospects. Nat. Rev. Drug Discov..

[9] Wadhwa S., Mumper R.J. (2015). Polymer-Drug Conjugates for Anticancer Drug Delivery. Crit. Rev. Ther. Drug Carrier Syst..

[10] Petros R.A., Desimone J.M. (2010). Strategies in the Design of Nanoparticles for Therapeutic Applications. Nat. Rev. Drug Discov..

[11] Chekkat N., Dahm G., Chardon E., Wantz M., Sitz J., Decossas M., Lambert O., Frisch B., Rubbiani R., Gasser G. (2016). N-Heterocyclic Carbene-Polyethylenimine Platinum Complexes with Potent in Vitro and in Vivo Antitumor Efficacy. Bioconjug. Chem..

[12] Torchilin V. (2011). Tumor Delivery of Macromolecular Drugs Based on the EPR Effect. Adv. Drug Deliv. Rev..

[13] Maeda H. (2017). Polymer Therapeutics and the EPR Effect. J. Drug Target..

[14] Fahira A.I., Abdulah R., Barliana M.I., Gatera V.A., Amalia R. (2022). Polyethyleneimine (PEI) as a Polymer-Based Co-Delivery System for Breast Cancer Therapy. Breast Cancer Targets Ther..

[15] Neuberg P., Kichler A. (2014). Recent Developments in Nucleic Acid Delivery with Polyethylenimines.

[16] Xu C., Wang P., Zhang J., Tian H., Park K., Chen X. (2015). Pulmonary Codelivery of Doxorubicin and SiRNA by PH-Sensitive Nanoparticles for Therapy of Metastatic Lung Cancer. Small.

[17] Zhou Z., Murdoch W.J., Shen Y. (2015). A Linear Polyethylenimine (LPEI) Drug Conjugate with Reversible Charge to Overcome Multidrug Resistance in Cancer Cells. Polymer.

[18] Wantz M., Bouché M., Dahm G., Chekkat N., Fournel S., Bellemin-Laponnaz S. (2018). N-Heterocyclic Carbene-Polyethyleneimine (PEI) Platinum Complexes Inducing Human Cancer Cell Death: Polymer Carrier Impact. Int. J. Mol. Sci..

[19] Neuzil J., Stantic M., Zobalova R., Chladova J., Wang X., Prochazka L., Dong L., Andera L., Ralph S.J. (2007). Tumour-Initiating Cells vs. Cancer “stem” Cells and CD133: What’s in the Name?. Biochem. Biophys. Res. Commun..

[20] Wu X.Z. (2008). Origin of Cancer Stem Cells: The Role of Self-Renewal and Differentiation. Ann. Surg. Oncol..

[21] Batlle E., Clevers H. (2017). Cancer Stem Cells Revisited. Nat. Med..

[22] Ayob A.Z., Ramasamy T.S. (2018). Cancer Stem Cells as Key Drivers of Tumour Progression. J. Biomed. Sci..

[23] Zhao J. (2016). Cancer Stem Cells and Chemoresistance: The Smartest Survives the Raid. Pharmacol. Ther..

[24] Abdullah L., Chow E. (2013). Chemoresistance in Cancer Stem Cells. Clin. Transl. Med..

[25] Najafi M., Mortezaee K., Majidpoor J. (2019). Cancer Stem Cell (CSC) Resistance Drivers. Life Sci..

[26] Tanida I. (2011). Autophagy Basics. Microbiol. Immunol..

[27] Mizushima N. (2007). Autophagy: Process and Function. Genes Dev..

[28] Virolle T. (2017). Cancer Stem Cells in Glioblastoma. Bull. Cancer.

[29] Seymour T., Nowak A., Kakulas F. (2015). Targeting Aggressive Cancer Stem Cells in Glioblastoma. Front. Oncol..

[30] Lamb R., Harrison H., Hulit J., Smith D.L., Lisanti M.P., Sotgia F. (2014). Mitochondria as New Therapeutic Targets for Eradicating Cancer Stem Cells: Quantitative Proteomics and Functional Validation via MCT1/2 Inhibition. Oncotarget.

[31] Jagust P., De Luxán-Delgado B., Parejo-Alonso B., Sancho P. (2019). Metabolism-Based Therapeutic Strategies Targeting Cancer Stem Cells. Front. Pharmacol..

[32] Farnie G., Sotgia F., Lisanti M.P. (2015). High Mitochondrial Mass Identifies a Sub-Population of Stem-like Cancer Cells That Are Chemo-Resistant. Oncotarget.

[33] Skoda J., Borankova K., Jansson P.J., Huang M.L.H., Veselska R., Richardson D.R. (2019). Pharmacological Targeting of Mitochondria in Cancer Stem Cells: An Ancient Organelle at the Crossroad of Novel Anti-Cancer Therapies. Pharmacol. Res..

[34] Liou G.Y. (2019). CD133 as a Regulator of Cancer Metastasis through the Cancer Stem Cells. Int. J. Biochem. Cell Biol..

[35] Thapa R., Wilson G.D. (2016). The Importance of CD44 as a Stem Cell Biomarker and Therapeutic Target in Cancer. Stem Cells Int..

[36] Garg A.D., Nowis D., Golab J., Vandenabeele P., Krysko D.V., Agostinis P. (2010). Immunogenic Cell Death, DAMPs and Anticancer Therapeutics: An Emerging Amalgamation. Biochim. Biophys. Acta—Rev. Cancer.

[37] Tesniere A., Panaretakis T., Kepp O., Apetoh L., Ghiringhelli F., Zitvogel L., Kroemer G. (2008). Molecular Characteristics of Immunogenic Cancer Cell Death. Cell Death Differ..

[38] Zhou J., Wang G., Chen Y., Wang H., Hua Y., Cai Z. (2019). Immunogenic Cell Death in Cancer Therapy: Present and Emerging Inducers. J. Cell. Mol. Med..

[39] Pandolfi F., Altamura S., Frosali S., Conti P. (2016). Key Role of DAMP in Inflammation, Cancer, and Tissue Repair. Clin. Ther..

[40] Brissault B., Kichler A., Guis C., Leborgne C., Danos O., Cheradame H. (2003). Synthesis of Linear Polyethylenimine Derivatives for DNA Transfection. Bioconjug. Chem..

[41] Campos B., Wan F., Farhadi M., Ernst A., Zeppernick F., Tagscherer K.E., Ahmadi R., Lohr J., Dictus C., Gdynia G. (2010). Differentiation Therapy Exerts Antitumor Effects on Stem-like Glioma Cells. Clin. Cancer Res..

[42] Verreault M., Schmitt C., Goldwirt L., Pelton K., Haidar S., Levasseur C., Guehennec J., Knoff D., Labussière M., Marie Y. (2016). Preclinical Efficacy of the MDM2 Inhibitor RG7112 in MDM2-Amplified and TP53 Wild-Type Glioblastomas. Clin. Cancer Res. Off. J. Am. Assoc. Cancer Res..

[43] Taciak B., Białasek M., Braniewska A., Sas Z., Sawicka P., Kiraga Ł., Rygiel T., Król M. (2018). Evaluation of Phenotypic and Functional Stability of RAW 264.7 Cell Line through Serial Passages. PLoS ONE.

[44] Podergajs N., Brekka N., Radlwimmer B., Herold-Mende C., Talasila K.M., Tiemann K., Rajcevic U., Lah T.T., Bjerkvig R., Miletic H. (2013). Expansive Growth of Two Glioblastoma Stem-like Cell Lines Is Mediated by BFGF and Not by EGF. Radiol. Oncol..

[45] McCartin C., Mathieu E., Dontenwill M., Herold-Mende C., Idbaih A., Bonfiglio A., Mauro M., Fournel S., Kichler A. (2022). An N-Heterocyclic Carbene Iridium(III) Complex as a Potent Anti-Cancer Stem Cell Therapeutic. Chem. Biol. Interact..

[46] Prabhakar N., Merisaari J., Le Joncour V., Peurla M., Karaman D.Ş., Casals E., Laakkonen P., Westermarck J., Rosenholm J.M. (2021). Circumventing Drug Treatment? Intrinsic Lethal Effects of Polyethyleneimine (PEI)-Functionalized Nanoparticles on Glioblastoma Cells Cultured in Stem Cell Conditions. Cancers.

[47] Knauer N., Arkhipova V., Li G., Hewera M., Pashkina E., Nguyen P.-H., Meschaninova M., Kozlov V., Zhang W., Croner R. (2022). In Vitro Validation of the Therapeutic Potential of Dendrimer-Based Nanoformulations against Tumor Stem Cells. Int. J. Mol. Sci..

[48] Zhang Q.F., Wang B., Yin D.X., Zhang J., Wu W.X., Yu Q.Y., Yu X.Q. (2014). Linear TACN-Based Cationic Polymers as Non-Viral Gene Vectors. RSC Adv..

[49] Kheraldine H., Rachid O., Habib A.M., Al Moustafa A.E., Benter I.F., Akhtar S. (2021). Emerging Innate Biological Properties of Nano-Drug Delivery Systems: A Focus on PAMAM Dendrimers and Their Clinical Potential. Adv. Drug Deliv. Rev..

[50] Jevprasesphant R., Penny J., Jalal R., Attwood D., McKeown N.B., D’Emanuele A. (2003). The Influence of Surface Modification on the Cytotoxicity of PAMAM Dendrimers. Int. J. Pharm..

[51] Fischer D., Li Y., Ahlemeyer B., Krieglstein J., Kissel T. (2003). In Vitro Cytotoxicity Testing of Polycations: Influence of Polymer Structure on Cell Viability and Hemolysis. Biomaterials.

[52] Beyerle A., Irmler M., Beckers J., Kissel T., Stoeger T. (2010). Toxicity Pathway Focused Gene Expression Profiling of PEI-Based Polymers for Pulmonary Applications. Mol. Pharm..

[53] Moghimi S.M., Symonds P., Murray J.C., Hunter A.C., Debska G., Szewczyk A. (2005). A Two-Stage Poly(Ethylenimine)-Mediated Cytotoxicity: Implications for Gene Transfer/Therapy. Mol. Ther..

[54] Grandinetti G., Ingle N.P., Reineke T.M. (2011). Interaction of Poly(Ethylenimine)-DNA Polyplexes with Mitochondria: Implications for a Mechanism of Cytotoxicity. Mol. Pharm..

[55] Novikoff A.B., Beaufay H., De Duve C. (1956). Electron Microscopy of Lysosome-Rich Fractions from Rat Liver. J. Cell Biol..

[56] Smith A.G., Macleod K.F. (2019). Autophagy, Cancer Stem Cells and Drug Resistance. J. Pathol..

[57] Arima Y., Nobusue H., Saya H. (2020). Targeting of Cancer Stem Cells by Differentiation Therapy. Cancer Sci..

[58] El-Gowily A.H., Abosheasha M.A. (2021). Differential Mechanisms of Autophagy in Cancer Stem Cells: Emphasizing Gastrointestinal Cancers. Cell Biochem. Funct..

[59] Mizushima N., Yoshimori T., Levine B. (2010). Methods in Mammalian Autophagy Research. Cell.

[60] Bik E., Mateuszuk L., Orleanska J., Baranska M., Chlopicki S., Majzner K. (2021). Chloroquine-Induced Accumulation of Autophagosomes and Lipids in the Endothelium. Int. J. Mol. Sci..

[61] Mauthe M., Orhon I., Rocchi C., Zhou X., Luhr M., Hijlkema K.J., Coppes R.P., Engedal N., Mari M., Reggiori F. (2018). Chloroquine Inhibits Autophagic Flux by Decreasing Autophagosome-Lysosome Fusion. Autophagy.

[62] Chikte S., Panchal N., Warnes G. (2014). Use of LysoTracker Dyes: A Flow Cytometric Study of Autophagy. Cytom. Part A.

[63] Lu S., Sung T., Lin N., Abraham R.T., Jessen B.A. (2017). Lysosomal Adaptation: How Cells Respond to Lysosomotropic Compounds. PLoS ONE.

[64] Kichler A., Leborgne C., Coeytaux E., Danos O. (2001). Polyethylenimine-Mediated Gene Delivery: A Mechanistic Study. J. Gene Med..

[65] Akinc A., Thomas M., Klibanov A.M., Langer R. (2005). Exploring Polyethylenimine-Mediated DNA Transfection and the Proton Sponge Hypothesis. J. Gene Med..

[66] Liu W.J., Ye L., Huang W.F., Guo L.J., Xu Z.G., Wu H.L., Yang C., Liu H.F. (2016). P62 Links the Autophagy Pathway and the Ubiqutin–Proteasome System upon Ubiquitinated Protein Degradation. Cell. Mol. Biol. Lett..

[67] Gao X., Yao L., Song Q., Zhu L., Xia Z., Xia H., Jiang X., Chen J., Chen H. (2011). The Association of Autophagy with Polyethylenimine-Induced Cytotoxity in Nephritic and Hepatic Cell Lines. Biomaterials.

[68] Lin C.W., Jan M.S., Kuo J.H.S., Hsu L.J., Lin Y.S. (2012). Protective Role of Autophagy in Branched Polyethylenimine (25K)-and Poly(L-Lysine) (30–70K)-Induced Cell Death. Eur. J. Pharm. Sci..

[69] Wang F., Salvati A., Boya P. (2018). Lysosome-Dependent Cell Death and Deregulated Autophagy Induced by Amine-Modified Polystyrene Nanoparticles. Open Biol..

[70] Pasquier B. (2016). Autophagy Inhibitors. Cell. Mol. Life Sci..

[71] Wang X., Lee J., Xie C. (2022). Autophagy Regulation on Cancer Stem Cell Maintenance, Metastasis, and Therapy Resistance. Cancers.

[72] Ryskalin L., Gaglione A., Limanaqi F., Biagioni F., Familiari P., Frati A., Esposito V., Fornai F. (2019). The Autophagy Status of Cancer Stem Cells in Gliobastoma Multiforme: From Cancer Promotion to Therapeutic Strategies. Int. J. Mol. Sci..

[73] Zhuang W., Long L., Zheng B., Ji W., Yang N., Zhang Q., Liang Z. (2012). Curcumin Promotes Differentiation of Glioma-Initiating Cells by Inducing Autophagy. Cancer Sci..

[74] Lei Y., Zhang D., Yu J., Dong H., Zhang J., Yang S., Yang S. (2017). Targeting Autophagy in Cancer Stem Cells as an Anticancer Therapy. Cancer Lett..

[75] Hao C., Liu G., Tian G. (2019). Autophagy Inhibition of Cancer Stem Cells Promotes the Efficacy of Cisplatin against Non-Small Cell Lung Carcinoma. Ther. Adv. Respir. Dis..

[76] Babaei G., Aziz S.G.G., Jaghi N.Z.Z. (2021). EMT, Cancer Stem Cells and Autophagy; The Three Main Axes of Metastasis. Biomed. Pharmacother..

[77] Gamrekelashvili J., Greten T.F., Korangy F. (2015). Immunogenicity of Necrotic Cell Death. Cell. Mol. Life Sci..

[78] Wang Y., Martins I., Ma Y., Kepp O., Galluzzi L., Kroemer G. (2013). Autophagy-Dependent ATP Release from Dying Cells via Lysosomal Exocytosis. Autophagy.

[79] Luo X., Qiu Y., Dinesh P., Gong W., Jiang L., Feng X., Li J., Jiang Y., Lei Y.L., Chen Q. (2021). The Functions of Autophagy at the Tumour-Immune Interface. J. Cell. Mol. Med..

[80] Wang Y.J., Fletcher R., Yu J., Zhang L. (2018). Immunogenic Effects of Chemotherapy-Induced Tumor Cell Death. Genes Dis..

