# Supplementary material for: Polyethylenimine, an Autophagy-Inducing Platinum-Carbene-Based Drug Carrier with Potent Toxicity towards Glioblastoma Cancer Stem Cells
Source: Cancers (Basel). 2022 Oct 15;14(20):5057. doi: 10.3390/cancers14205057 (PMC9599868; doi:10.3390/cancers14205057)
Supplement: Supplementary file 1 [file cancers-14-05057-s001.zip › cancers-1959404-supplementary.pdf]

Supplementary information

Polyethylenimine, an Autophagy-Inducing Platinum-Carbene-Based Drug Carrier with Potent Toxicity
towards Glioblastoma Cancer Stem Cells

Conor McCartin [1], Candice Dussouillez [1], Chloé Bernhard [2], Eric Mathieu [3,4], Juliette Blumberger [1], Monique Dontenwill [2], Christel Herold-Mende [5], Ahmed Idbaih [6], Philippe Lavalle [3,4], Stéphane Bellemin-Laponnaz [7], Antoine Kichler [1,*] and Sylvie Fournel [1,*]

[1] 3Bio Team, CAMB UMR7199 CNRS, Faculté de Pharmacie, University of Strasbourg, 67401 Ilkirch, France
[2] Laboratoire de Bioimagerie et Pathologies UMR CNRS 7021 (LBP), Faculté de Pharmacie, University of Strasbourg, 67401 Illkirch, France
[3] Institut National de la Santé et de la Recherche Médicale, Inserm UMR_S 1121 Biomaterials and Bioengineering, 67085 Strasbourg, France
[4] Faculté de Chirurgie Dentaire de Strasbourg, University of Strasbourg, 67000 Strasbourg, France
[5] Division of Neurosurgical Research, Department of Neurosurgery, University of Heidelberg, 69117 Heidelberg, Germany
[6] AP-HP, Institut du Cerveau—Paris Brain Institute—ICM, Inserm, CNRS, Hôpitaux Universitaires La Pitié Salpêtrière—Charles Foix, DMU Neurosciences, Service de Neurologie 2-Mazarin, Sorbonne University, 75013 Paris, France
[7] Institut de Physique et Chimie des Matériaux de Strasbourg (IPCMS) UMR7504 CNRS, University of Strasbourg, 67034 Strasbourg, France
* Correspondence: kichler@unistra.fr (A.K.); s.fournel@unistra.fr (S.F.)

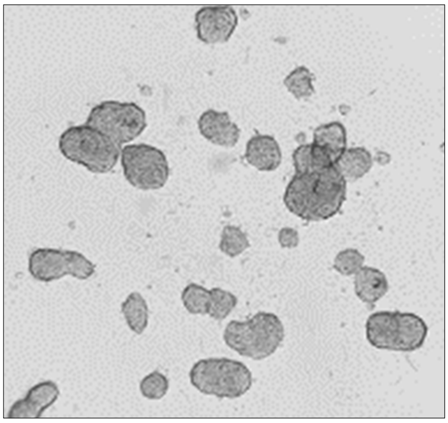

Figure S1. **DPSC spheroid cell culture.** Inverted light microscope image of DPSC spheroids grown in U-bottomed suspension 96 well culture dishes for 24 h before imaging at 4x*10x = 400x magnification using an Axio Vert A1 inverted light microscope (Zeiss) coupled to a ProgRes C5 cool (Jenoptik) camera.

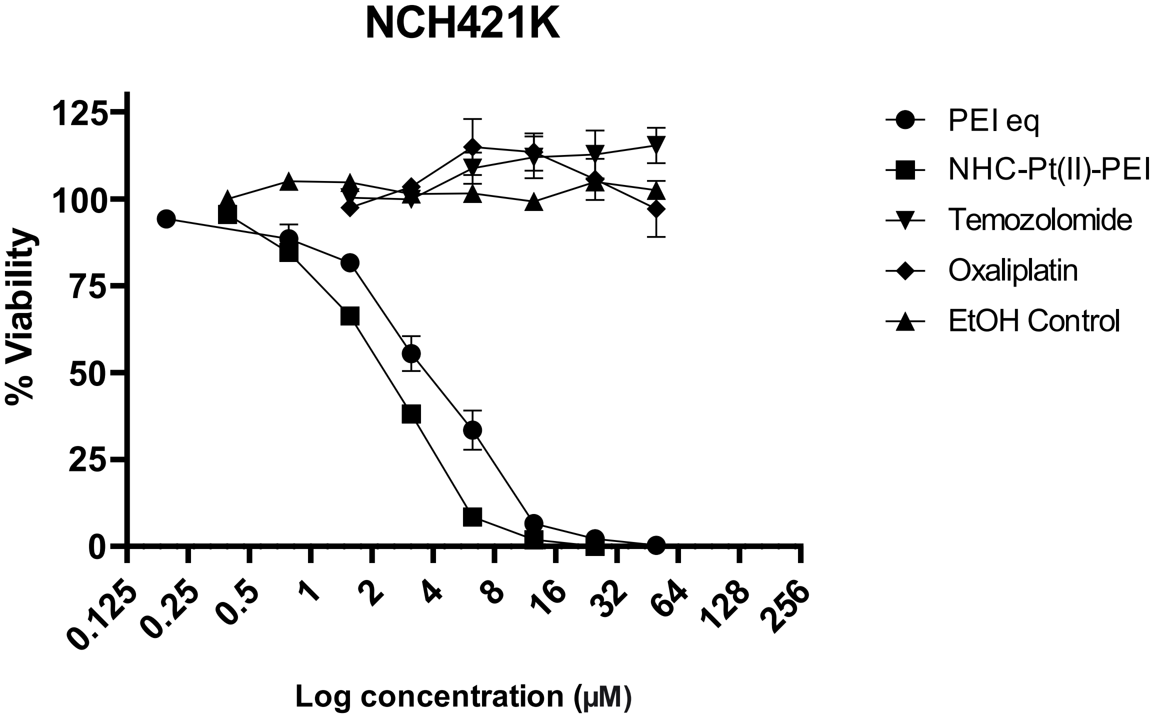

Figure S2. **IC_{50} for the GSC cell line NCH421K.** CelltiterGlo 3D viability dose-response of 24 h treated NCH421K cells. Values represent the mean of at least n=3 independent replicates. Error bars represent +/- one SEM.

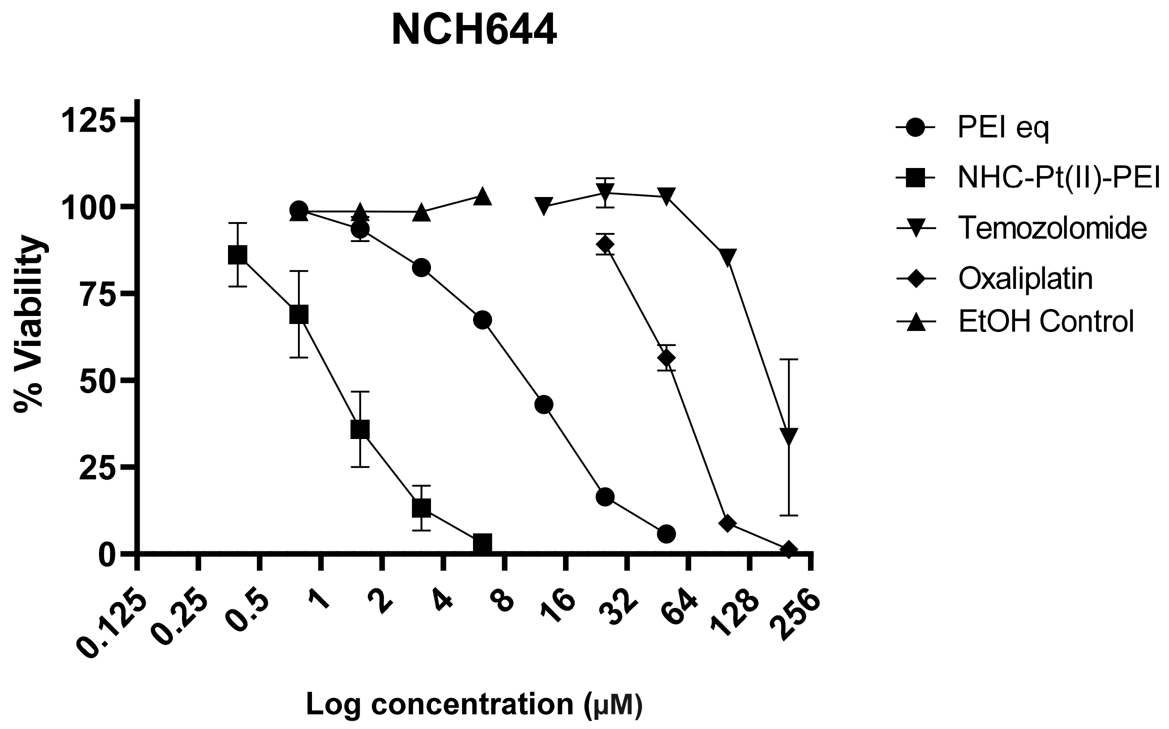

Figure S3. **IC$_{50}$ for the GSC cell line NCH644.** CelltiterGlo 3D viability dose-response of 24 h treated NCH644 cells. Values represent the mean of at least n=3 independent replicates. Error bars represent +/- one SEM.

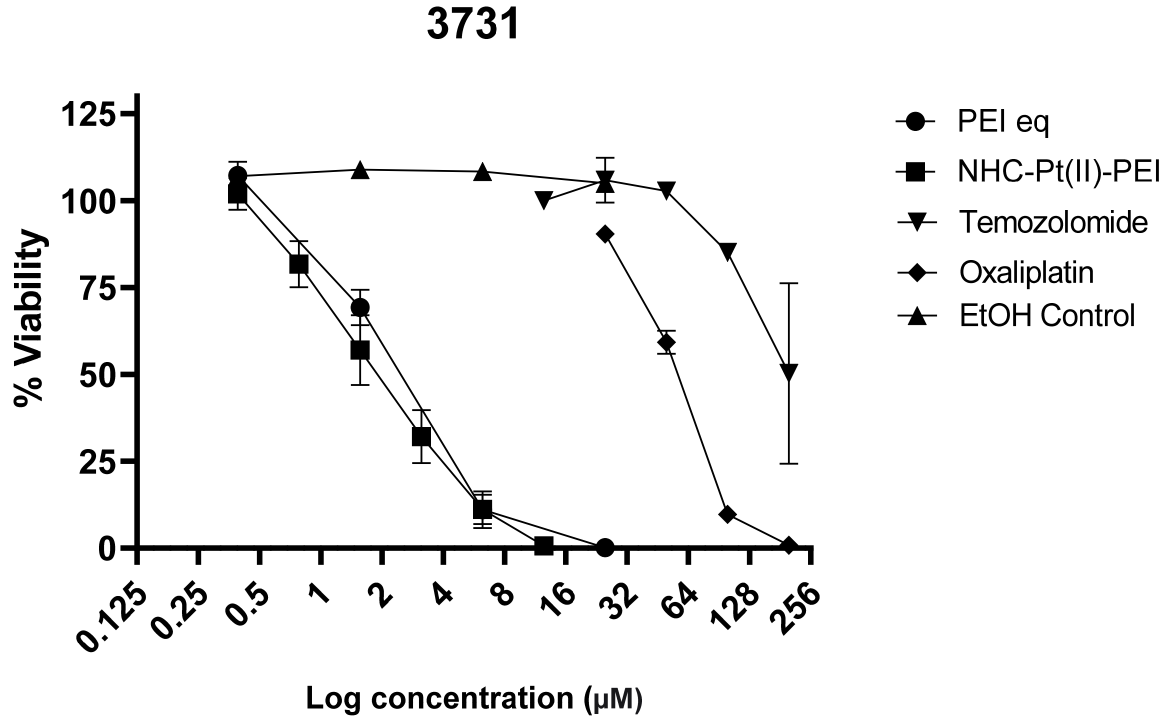

Figure S4. **IC50 for the GSC cell line 3731.** CelltiterGlo 3D viability dose-response of 24 h treated 3731 cells. Values represent the mean of at least n=3 independent replicates. Error bars represent +/- one SEM

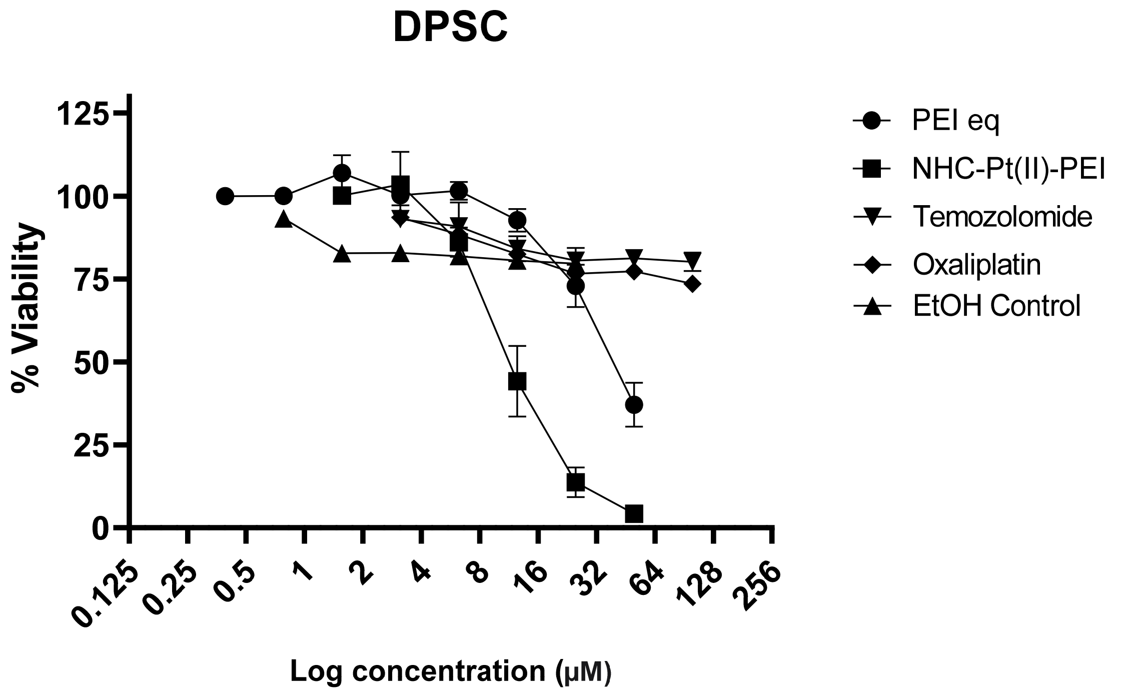

Figure S5. **IC50 for the non-cancerous stem cells, DPSCs.** CelltiterGlo 3D viability dose-response of 24 h treated DPSC cells. Values represent the mean of at least n=3 independent replicates. Error bars represent +/- one SEM.

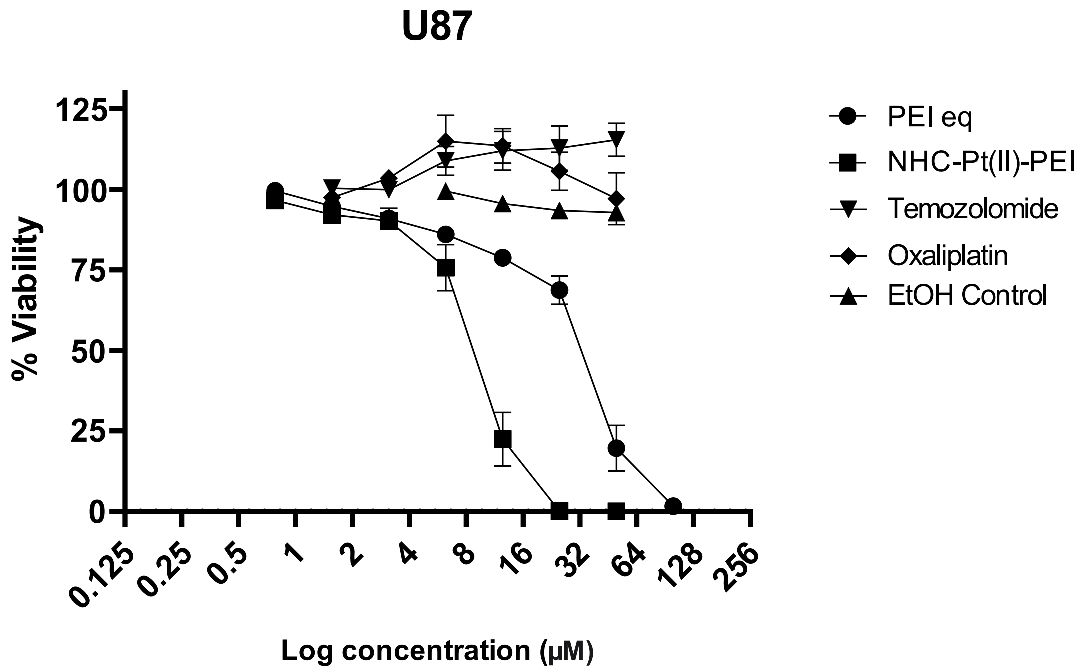

Figure S6. IC50 for the non-stem glioma cell line U87-MG. CelltiterGlo 3D viability dose-response of 24 h treated U87 cells. Values represent the mean of at least n=3 independent replicates. Error bars represent +/- one SEM.

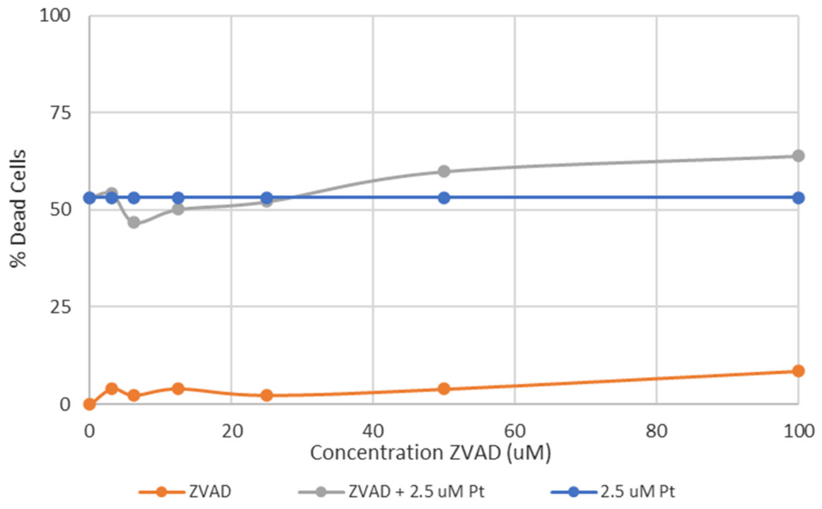

Figure S7. Cell death proportion of 24 h NHC-Pt(II)-PEI and zVAD-fmk co-treated NCH421K cells. The proportion of propidium iodide positive (dead) cells measured by flow cytometry following 24 h treatment with 2.5 µM NHC-Pt(II)-PEI (noted Pt), a cascade of concentrations of zVAD-fmk (noted ZCAD) or 2.5 µM NHC-Pt(II)-PEI + ZVAD.

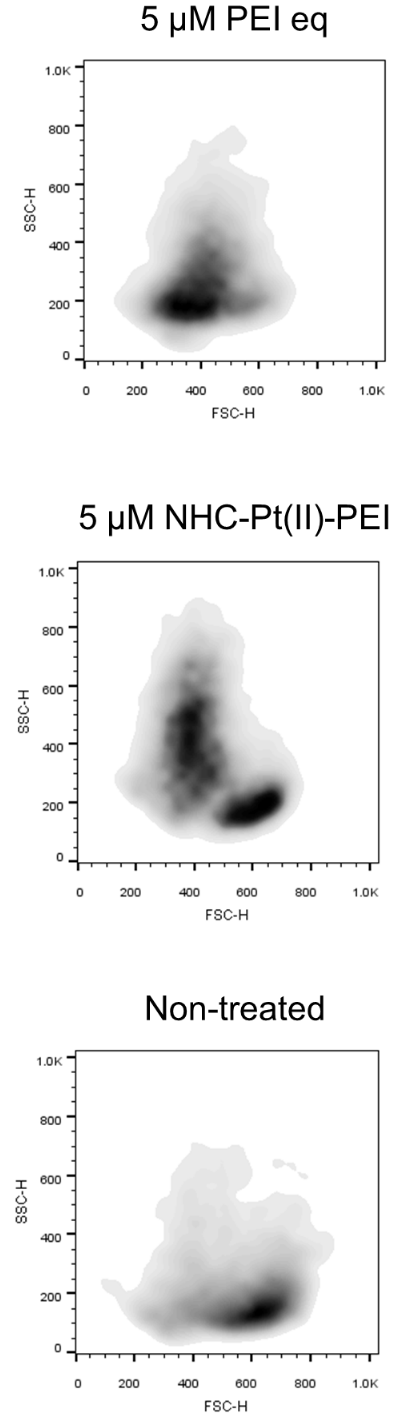

Figure S8. **The granularity of 24 h 5 µM PEI eq and NHC-Pt(II)-PEI treated NCH421K spheroids.** Flow cytometry FSC and SSC dot plots (FlowJo) of treated cells.

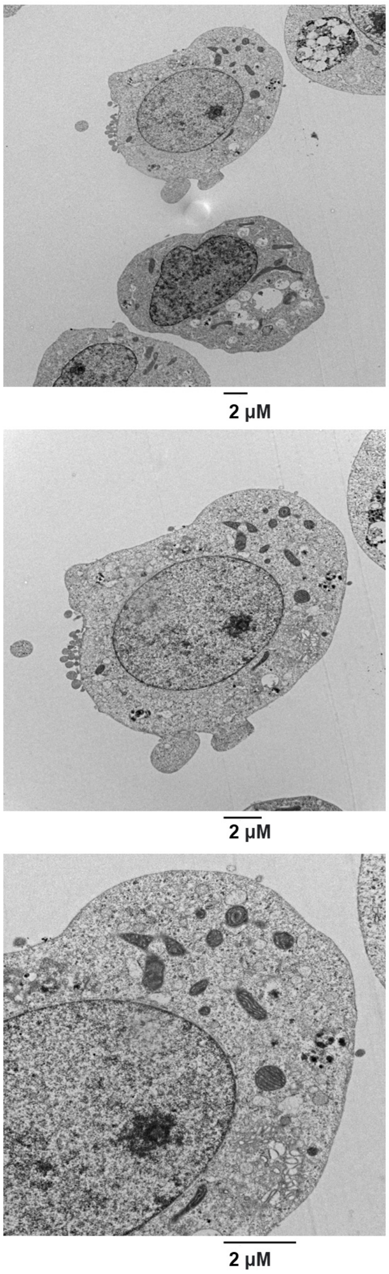

Figure S9. **Transmission electron microscopy of 6 h 60 µM chloroquine treated NCH421K cells.** Top = 5000x magnification. Middle = 8000x magnification. Bottom = 15,000x magnification.

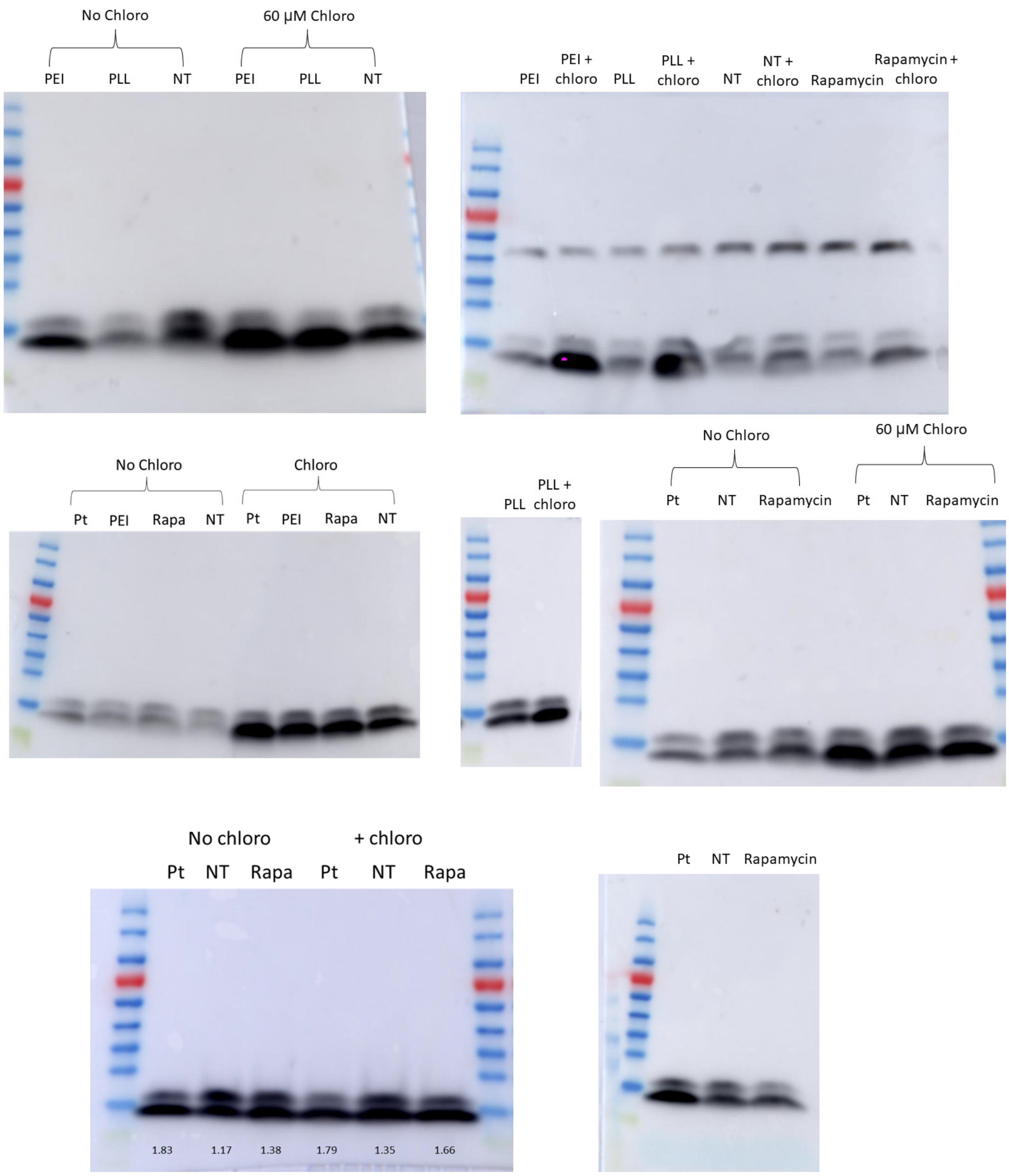

Figure S10. **Unedited LC3 western blots.** All treatments 6 h except chloroquine (3 h). Pt = NHC-Pt(II)-PEI. Rapamycin (Rapa) = 10 µM. Poly-L-Lysine (PLL) = 5 µM Pt eq (294 nM polymer). Polyethylenimine (PEI) = 5 µM Pt eq (294 nM polymer). Non-treated = NT.

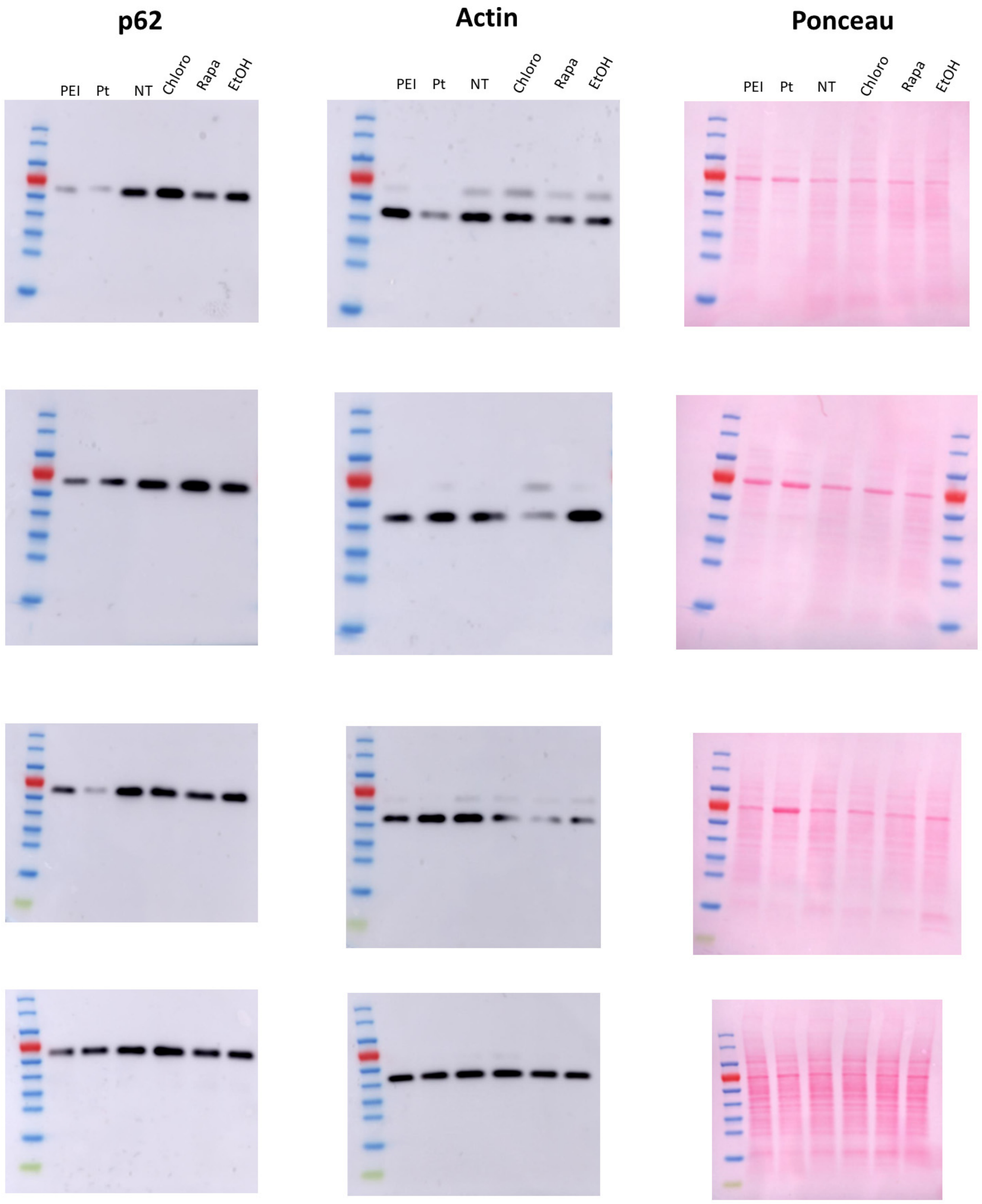

Figure S11. Unedited p62 western blots. Pt (NHC-Pt(II)-PEI) = 5 µM. Polyethylenimine (PEI) = 5 µM Pt eq (294 nM polymer). Non-treated = NT. Chloro (chloroquine) = 60 µM. Rapamycin (Rapa) = 10 µM. Ethanol control (EtOH) = Equivalent EtOH quantity corresponding to NHC-Pt(II)-PEI condition. All treatments 6 h.

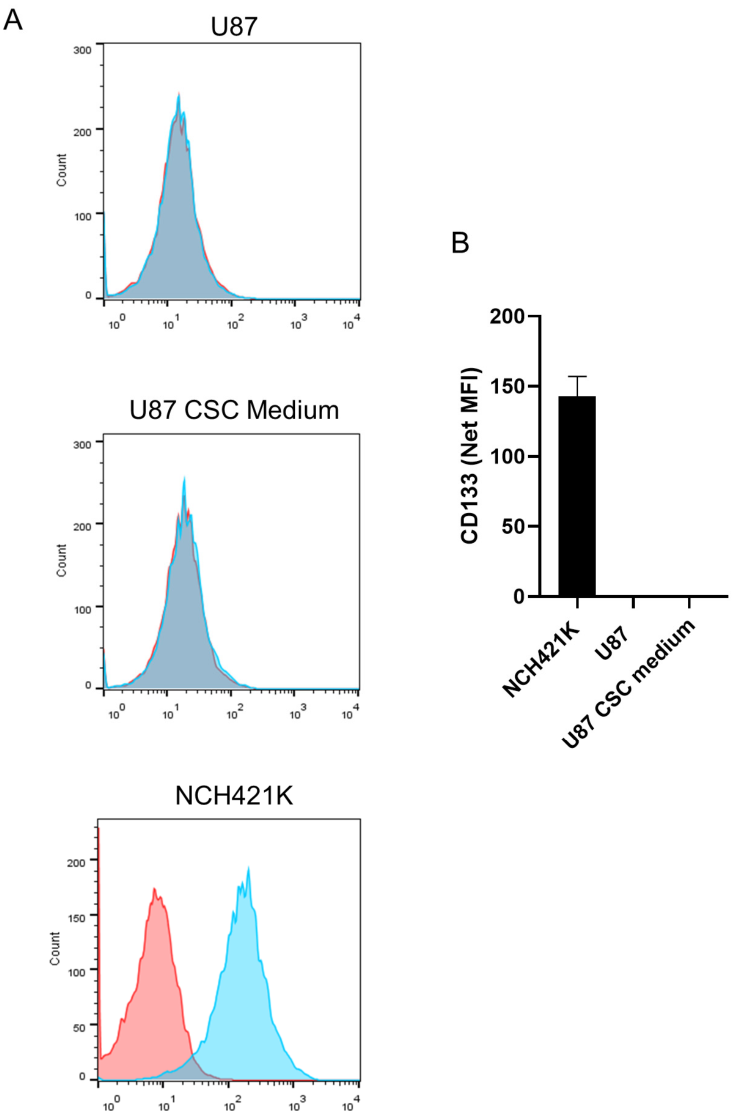

Figure S12. **U87 CD133 expression.** A) Representative flow cytometry histograms (FlowJo) of CD133 expression on U87 cells, U87 cells cultured for 24 h in CSC medium, and NCH421K cell. Red = isotype. Blue = CD133. B) Histograms of net MFI (geometric mean fluorescence intensity) (MFI CD133 – MFI isotype) CD133 expression. Values represent the mean of n=3 independent experiments ± one SEM.
